# Phase memory of optical vortex beams

**DOI:** 10.1038/s41598-022-14074-4

**Published:** 2022-06-21

**Authors:** Mahdi Eshaghi, Cristian Hernando Acevedo, Mahed Batarseh, José Rafael Guzman-Sepulveda, Aristide Dogariu

**Affiliations:** 1 CREOL, The College of Optics and Photonics, University of Central Florida, 4304 Scorpius St, Orlando, FL 32816 USA; 2 Present Address: Center for Research and Advanced Studies, National Polytechnic Institute, 66600 Apodaca, Nuevo Leon Mexico

**Keywords:** Optics and photonics, Physics

## Abstract

Optical vortex beams are under considerable scrutiny due to their demonstrated potential for applications ranging from quantum optics to optical communications and from material processing to particle trapping. However, upon interaction with inhomogeneous material systems, their deterministic properties are altered. The way these structured beams are affected by different levels of disturbances is critical for their uses. Here, for the first time, we quantify the degradation of perfect optical vortex beams after their interaction with localized random media. We developed an analytical model that (1) describes how the spatial correlation and the phase variance of disturbance affect the phase distribution across the vortex beams and (2) establishes the regimes of randomness for which the beams maintain the memory of their initial vorticity. Systematic numerical simulations and controlled experiments demonstrate the extent of this memory effect for beams with different vorticity indices.

## Introduction

When propagating in free space, the electromagnetic fields could contain robust phase singularities. In these points the amplitude vanishes and the phase cannot be determined. This field property was called "screw dislocation" in analogy to crystal lattice defects^1,2^. When the field propagates, the lines of constant phases around a singularity trace out a spiral that is mathematically similar to superfluid vortices, which inspired the term "optical vortex"^3^. To some degree, the vortices embedded in a light beam, termed "optical vortex" beam (OV), act as charged particles so that they may rotate around the beam axis, repel and attract each other, and created or annihilated in dipole pairs^4,5^. Vortices are characterized by their "singularity strength" or "topological charge" (TC)^6^, which denotes the number of twists of the phase front. Since the beginning, the properties of optical vortices attracted a significant attention, especially in the case of random fields^7–10^, where it was shown that their number density can be pretty high. In fact, in the case of so-called fully developed speckle fields, one optical vortex (zero amplitude) accompanies one speckle spot (maximum amplitude)^7,11–13^.

Detecting the phase singularities where "spiral" and "fork" shaped fringes occur usually requires collinear or tilted interferometric techniques^8,14–16^. Non-interferometric techniques can also be used when studying the far-field diffraction of OVs by different elements^17–20^. Note that all these methods determine the TC of a singularity irrespective of the spatial distribution of different local vortices. This problem can be addressed by the simultaneous measurement of the beam’s amplitude and phase^21,22^.

The light field of an OV carries angular momentum which always contain an orbital (OAM) and a spin (SAM) component, which depends on the state of polarization^23–25^. The OAM originates from the light surrounding the singularity; the line of singularity does not carry energy and it has no momentum^26^. In the case of OVs embedded into azimuthally symmetric beams, such as Bessel^27^, Laguerre–Gauss^28^, and Bessel-Gauss^29^ beams, the TC and OAM have the same value. However, there are many other cases where these two parameters have different values^6,30–33^ and, in general, there is no direct relationship between them.

Several reports conjectured that, because they carry OAM, OVs propagate through optical turbulence with less distortion than conventional Gaussian beams^34–36^. Other experimental studies have shown that, in comparison with Gaussian beams, OVs are less stable for small propagation distances but they become more resilient over considerable distances^37^. Indeed, there are different criteria for beam stability. Sometimes maintaining TC is a critical characteristic while in other situations the so-called scintillation index can be an appropriate parameter. It has been shown that TC is a robust quantity since it can be transmitted over significant distance in the presence of atmospheric turbulence, which means it can be used to encode information in free-space optical communications^38^. On the other hand, the OAM modes span an infinite-dimensional basis, which can be used to send photon-level information^39^. Nevertheless, after passing through different types of turbulent atmospheres, the core of a vortex beam wanders away from its origin, which seriously hinders such applications^38,40–42^. Therefore, describing the statistical properties of the phase dislocations is a topic that has received a considerable attention^34,40,43–49^. A few works addressed the possibility to recover the vorticity of OVs after scattering by random phase screens^50–53^. However, most of these reports are rather qualitative and, moreover, there is no clear description of what is meant by perturbative media such as "diffusers" or "ground glasses."

Here, we provide a quantitative description of the OV degradation after the interaction with an inhomogeneous medium, which is characterized statistically by its "spatial correlation length" and the "variance" of phase randomness. In particular, we address the case of a "perfect optical vortex beam" (POV) for which the size of the beam is independent of its phase structure^54–56^. In the following, we will evaluate the extent of vortex memory, i.e., the range of randomness for which the initial vorticity can still be recovered. In doing so, we will develop an exact statistical relationship between the TC and the OAM modes for the perturbed field, such that one parameter can be determined from measurements of the other. The vortex memory range will be first established theoretically and then demonstrated experimentally.

## Theoretical model

Let us start from the description of a Bessel-Gauss beam^29^

1 $$E_{1} \left( {\rho ,\phi } \right) = {\text{J}}_{m} \left( {k_{r} \rho } \right){ }e^{im\phi } e^{{ - \rho^{2} /\omega_{g}^{2} }} ,$$

where $m$ is the TC, ${\text{J}}_{m}$ is the Bessel function of the first kind and order $m$, $\omega_{g}$ is the beam waist of the Gaussian beam used to generate the Bessel-Gauss beam, and $\left( {\rho ,\phi } \right)$ are the corresponding polar coordinates. The Fourier transformation of $E_{1} \left( {\rho ,\phi } \right)$ leads to the far-field distribution

2 $$E_{2} \left( {r,\theta } \right) = i^{m - 1} \left( {\omega_{g} /\omega_{0} } \right)e^{im\theta } e^{{ - \left( {r^{2} + r_{0}^{2} } \right)/\omega_{0}^{2} }} \rm I_{m} \left( {2r_{0} r/\omega_{0}^{2} } \right)$$

where ${\text{I}}_{m}$ is the modified Bessel function of the first kind of order $m$, $2\omega_{0}$ and $r_{0}$ are the ring width and radius, respectively, and $\left( {r,\theta } \right)$ are the polar coordinates in this plane. When the argument of ${\text{I}}_{m}$ is sufficiently large, this function behaves as an exponential, and Eq. (2) describes a so-called "perfect optical vortex" beam (POV)^56^

3 $$E_{2} \left( {r,\theta } \right) \cong E_{0} e^{{im\theta }} ~\updelta \left( {r - r_{0} } \right)$$

where $E_{0} = i^{m - 1} \left( {\omega_{g} /\omega_{0} } \right)$ is a constant complex coefficient.

This beam is incident on a phase screen $T\left( {r,\theta } \right) = {\text{exp}}\left( {i\psi \left( {r,\theta } \right)} \right)$ where $\psi \left( {r,\theta } \right) = \psi_{0} + \psi^{\prime } \left( {r,\theta } \right)$ is a random function of mean $\psi_{0}$. At a distance $z$ in the far-field, the distribution of the perturbed field can be written in polar coordinates as

4 $$E_{3} \left( {\rho ,\phi } \right) = \left( {E_{0} /N} \right)\mathop \sum \limits_{n = 1}^{N} e^{{i\psi \prime_{{\left( {n;r = r_{0} } \right)}} }} e^{{iR_{n} }}$$

where $R_{n} = \psi_{0} + m\theta_{n} - \mu r_{0} \rho {\text{cos}}\left( {\phi - \theta_{n} } \right)$ is a deterministic function of $n$ and $\mu = \pi /\lambda z$. Along the POV circumference, $N$ represents the number of independent random phase elements such that $\theta_{n} = n\left( {2\pi /N} \right)$ for $n \in \left[ {1,N} \right]$. To simplify the notation, we will replace $\psi_{{\left( {n;r = r_{0} } \right)}}^{\prime }$ by $\psi_{n}^{\prime }$ and $E_{3} \left( {\rho ,\phi } \right)$ by $E\left( {\rho ,\phi } \right)$.

In describing the random phase screen, we will define a "spatial correlation length" that encompasses $d$ elements with the same phase. When $N \gg d$, this correlation length is spatially invariant along the circumference of the ring. We will consider a Gaussian distribution for the probability density function $p\left( {\psi^{\prime } } \right)$ of random phases over an adjustable range $\left[ { - \alpha \pi ,\alpha \pi } \right]$. Thus, the normalized probability density function is $p\left( {\psi^{\prime } } \right) = \left( {\sqrt {2\pi s^{2} } {\text{erf}}\left( {\alpha \pi /\sqrt {2s^{2} } } \right)} \right)^{ - 1} {\text{exp}}\left( { - \psi^{\prime 2} /2s^{2} } \right)$ where $s$ is the standard deviation and ${\text{erf}}$ denotes the error function. Varying the parameter $\alpha$ permits accounting for an entire range of phase distribution functions from Dirac delta $\left( {s \to 0} \right)$ to uniform $\left( {s \to \infty } \right)$ distributions. Throughout this manuscript, we refer to $\alpha$ as the "phase variance."

As detailed in Supplementary Material 1, the field $E\left( {\rho ,\phi } \right)$ can be expanded on a vorticity basis as

5 $$E\left( {\rho ,\phi } \right) = \mathop \sum \limits_{k = - \infty }^{\infty } H\left( {k;\rho } \right){ }e^{ik\phi }$$

where each complex coefficient $H\left( {k;\rho } \right)$ is defined as

6 $$H\left( {k;\rho } \right) = {{\upphi}} \cdot A\mathop \sum \limits_{n = 1}^{N/d} e^{{i\psi_{n}^{\prime } }} e^{{i\left[ {m - k} \right]\theta_{{\left( {n - 1} \right)d}} }}$$

with ${{\upphi}} = \left( { - i} \right)^{\left| k \right|} e^{{i\left[ {m - k} \right]\frac{d + 1}{N}\pi + i\psi_{0} }}$ and $$A = {\text{J}}_{\left| k \right|} \left( {\mu r_{0} \rho } \right){\text{ sinc}}\left( {\left[ {m - k} \right]\left( {d/N} \right)\pi } \right)$$ being the phase and amplitude terms that factorize out of the summation. Based on the distribution of $$p\left( {\psi^{\prime } } \right)$$, one can find the statistical properties of each complex coefficient $$H\left( {k;\rho } \right)$$, including the average and all other higher-order moments. Knowing these statistical properties, one can study in detail the behavior of the effective TC and the OAM modes in response to changes in the randomness defined by its correlation length and phase variance. To this end, we will evaluate these parameters over a circular contour of arbitrary radius $$\rho_{0}$$ which is centered on the optical axis. Inside this contour, we can calculate the weight $$V^{k} = \langle\left| {H\left( {k;\rho } \right)} \right|^{2} \rangle|_{{\rho = \rho_{0} }}$$ for vorticity mode of order $$k$$^57^. The corresponding OAM mode can then be gauged as $$L^{k} = \mathop \smallint \limits_{0}^{{\rho_{0} }} \langle\left| {H\left( {k;\rho } \right)} \right|^{2}\rangle \rho d\rho$$^58^. The most probable values for the vorticity and OAM modes are further determined by evaluating the statistical average $$\langle x \rangle = \mathop \sum \limits_{i} p_{i} x_{i} /\mathop \sum \limits_{i} p_{i}$$ where $$x$$ can be either $$V$$ or $$L$$. In the end, we establish a statistical relation between each vorticity mode $$L^{k}$$ and the corresponding OAM mode $$V^{k}$$ as

7 $$L^{k} /V^{k} = \left( {\rho_{0}^{2} /2} \right)\left\{ {1 - {\text{J}}_{\left| k \right| - 1} \left( {\mu r_{0} \rho_{0} } \right){\text{J}}_{\left| k \right| + 1} \left( {\mu r_{0} \rho_{0} } \right)/{\text{J}}_{\left| k \right|}^{2} \left( {\mu r_{0} \rho_{0} } \right)} \right\}$$

which is independent of the properties of scattering media. A step-by-step derivation of these parameters is included in the Supplemental Materials 1, together with the closed analytical form for the weights of each TC and OAM modes inside the circular contour.

## Results and discussion

The overall goal is to understand how a number $\left| m \right|$ of local vortices evolve after a beam with initial vorticity $m$ is perturbed by the interaction with a random phase screen^5^. For this, one needs to define a suitable observation scale such that vortices close to the optical axis can be tracked appropriately, as well as a clear criterion so that comparison among different cases is possible.

The extent of "vortex memory" is determined by the rate at which the initial vorticity $m$ vanishes when the phase variance of randomness varies for a determined correlation length. A quantitative criterion can be established by defining, for instance, the regime of randomness for which the average vorticity remains larger than $m - 1$. Up to this point, the field has maintained a certain memory of its initial conditions. Then, by increasing the disturbance, the mean vorticity continues to decay from $m - 1$ to $0$, because the perturbed field evolves towards a stochastically homogenous and isotropic field (fully-developed speckle pattern). In the initial case, the field is deterministic and can be described either locally or globally. However, in the final case of fully-developed speckle a global description is meaningless and one has to appeal to local distributions of amplitude and phase. The behavior of this transition can also be described using the memory effect.

It is known that the mean number density of dislocations inside a fully-developed speckle pattern equals $1/2a_{\text{coh}}$ where $a_{\text{coh}} = \lambda^{2} z^{2} /\pi r_{0}^{2}$ is the average coherence area^12^. This means that, within a circle of radius $\rho_{0} = 2\sqrt {a_{\text{coh}} { }/{\uppi }}$ such that $\mu r_{0} \rho_{0} = 2$, there are, on average, two vortices of opposite handedness. In the following, we will use $\rho_{0}$ as to limit the spatial extent of our analysis. We chose this because the distance between a local vortex and its closest neighbor with same-handedness can vary in the range $\left[ {0,2\rho_{0} } \right]$. From a practical viewpoint, a topological charge larger than unity within a circle of radius $\rho_{0}$ indicates, locally, a high-order vorticity, so that the phase can be described and measured globally. In such case, there is no need for measuring the spatial distribution of the complex field.

**Figure 1:**
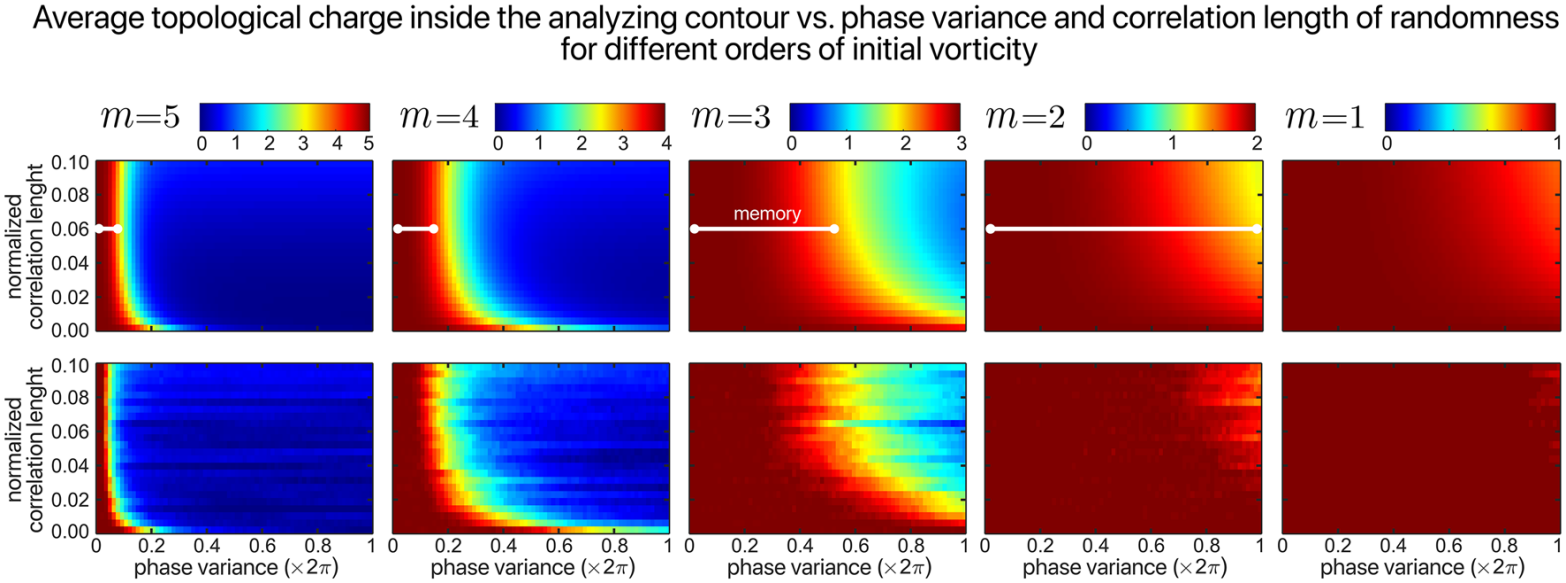
Analytical (top row) and numerical (bottom row) illustration of the phase memory in optical vortex beams. The color scale represents the vorticity evaluated over a circular contour of radius $\rho_{0} = 2\sqrt {a_{coh} /\pi }$ for the case where the randomness is Gaussian distributed with $s/\alpha = 0.3\pi$. The spatial correlation length of randomness is normalized over the circumference of the POV. The numerical simulation data reflects an average over 50 realizations of randomness. The white lines indicate the range of phase variance where the initial vorticity can be recovered, i.e., the extent of "vortex memory".

We conducted a detailed numerical simulation of different interaction regimes. After generating the stochastic field based on the initial beam structure and randomness properties, we implemented an algorithm to find the location of vortices, as well as their handedness. By changing the randomness parameters, we tracked the position of main vortices, which are generated due to initial vorticity and then evaluate the total charge inside the chosen closed contour. Typical results are illustrated in Fig. 1 where a quantitative comparison between the analytical predications and the numerical calculations is presented. As mentioned previously, the evaluations are performed over a circle of radius $\rho_{0}$. The probability density function in this case is a Gaussian distribution with standard deviation $s = \alpha \times 0.3\pi$ in the range $\left[ { - \alpha \pi ,\alpha \pi } \right]$, for different values of the correlation length $d$. The details regarding the numerical simulation can be found in Supplementary Material 2.

The first important conclusion relates to the influence of the spatial correlation length. Its effect on the effective topological charge is not linear as randomness with higher spatial correlation distorts the phase structure more dramatically. Our model accounts for the configuration of vortices generated out of the initial vortex located on the optical axis. In the case of vortex beam illumination, the speckles start forming along the circumference of the rings of light. Now, by increasing the correlation length while keeping the phase variance unchanged, the speckles shrink. Because each of the vortices connect to one undeveloped speckle, by decreasing the size of speckles, the separation between the vortices around optical axis increases. This is the reason why the TC inside the area of consideration drops faster when the correlation length increases.

Moving to the next conclusion, we note that in the present case, the illuminated area is constant or, in other words, the parameter $N$ in our model does not change. Therefore, by modifying the spatial correlation length, both the number and the type (or size) of the disturbing elements change such that their product is constant. Now, the model applies to the practical situation where the number of independent disturbing elements is always very large such that the central limit theorem applies. In these circumstances, the absolute number of random elements does not matter. The effects we observe are, therefore, the result of changes in the size of the independent scattering elements.

Second, we see that under similar conditions of phase disturbance, fields with higher vorticity decorrelate faster. This means that when the number of bunched local vortices with the same handedness increases, they tend to wander more rapidly. This can be explained by a simple analogy where local vortices are seen as interacting particles with positive (or negative) charges according to their handedness. The same sign charges repel each other stronger when more of them are gathered within a finite space. In addition, it can be observed that once $\left| m \right|$ vortices are being created, there is no preferential way in which they diffuse from the center. A somewhat similar behavior is observed when vortex beams propagate through turbulence and the intensity perturbations are analyzed in terms of OAM modal composition^49^.

The origin of "phase memory" is the processes of field randomization itself. When the excitation field is a beam with robust vortex structure, the effect of the scattering layer is to feed energy into the dark spatial regions of the incident field such that new speckles emerge in the initially dark regions. This is quite different from how randomness acts on, for instance, an initially uniform phase distribution when regions of decreasing intensity are newly created. In this case the effect of scattering is to disperse the energy from the region of interest.

**Figure 2:**
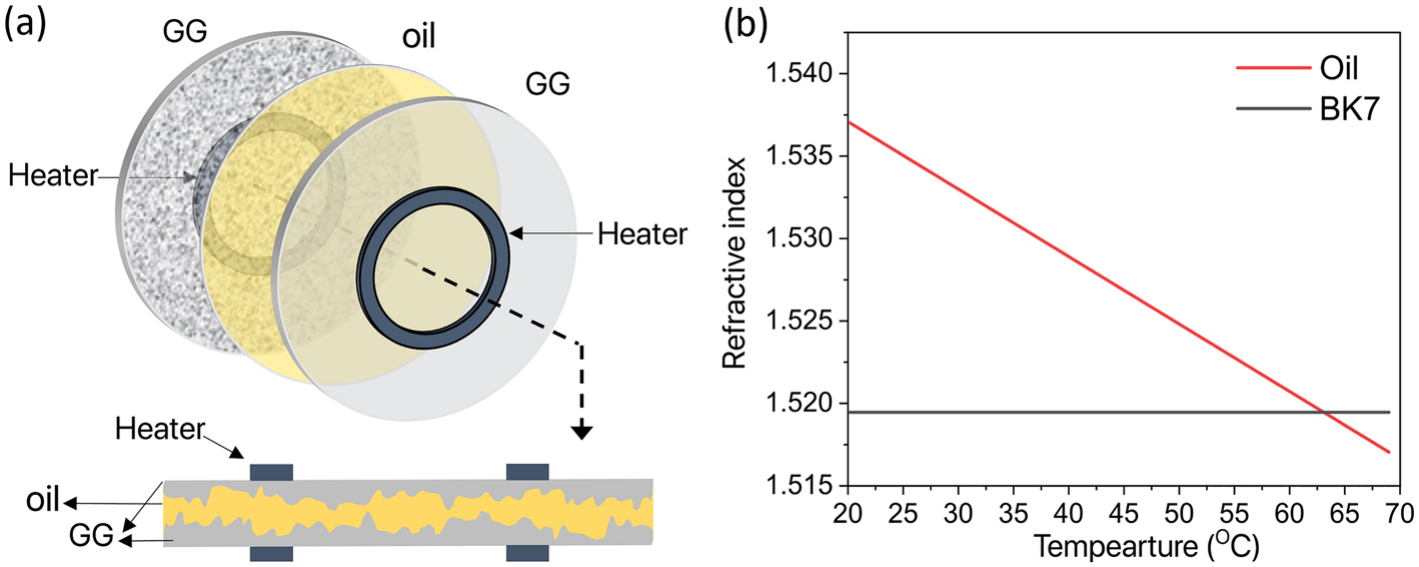
Tunable phase screen (TPS). (**a**) Schematic and cross-sections of TPS comprising two similar ground glasses (GG), a thin layer of refractive index oil, and two ring-shape heaters. (**b**) Change of the refractive index of the oil as temperature increases in comparison to the refractive index of glass (BK7) at λ = 532 nm.

We have also conducted an experimental demonstration where a "tunable phase screen" (TPS) was used to probe a range of phase variance of randomness. This device incorporates two ground glasses (GG) with similar statistical properties and a thin layer of variable-refractive index oil in between. By adjusting the oil temperature, the refractive index mismatch between oil and glass can be varied at will therefore controlling the randomness phase variance. Figure 2a shows the schematic of the TPS. At room temperature, the refractive index mismatch between glass and oil is maximum, which leads to the maximum strength of scattering. Increasing the temperature reduces the refractive index of the oil and effectively reduces the scattering strength. The evolution of refractive index with temperature is shown in Fig. 2b where the intersection of the two lines denotes the matching condition corresponding to minimal scattering.

If one knows how the phase changes in response to temperature variations, then a quantitative comparison between the model and experiment can be performed. To this end, we have fully characterized the roughness distribution. We evaluated its auto-correlation function and determined the effective distribution of phase and spatial correlation length of our TPS for a specific value of the oil's refractive index. All details of this procedure can be found in Supplementary Material 3.

**Figure 3:**
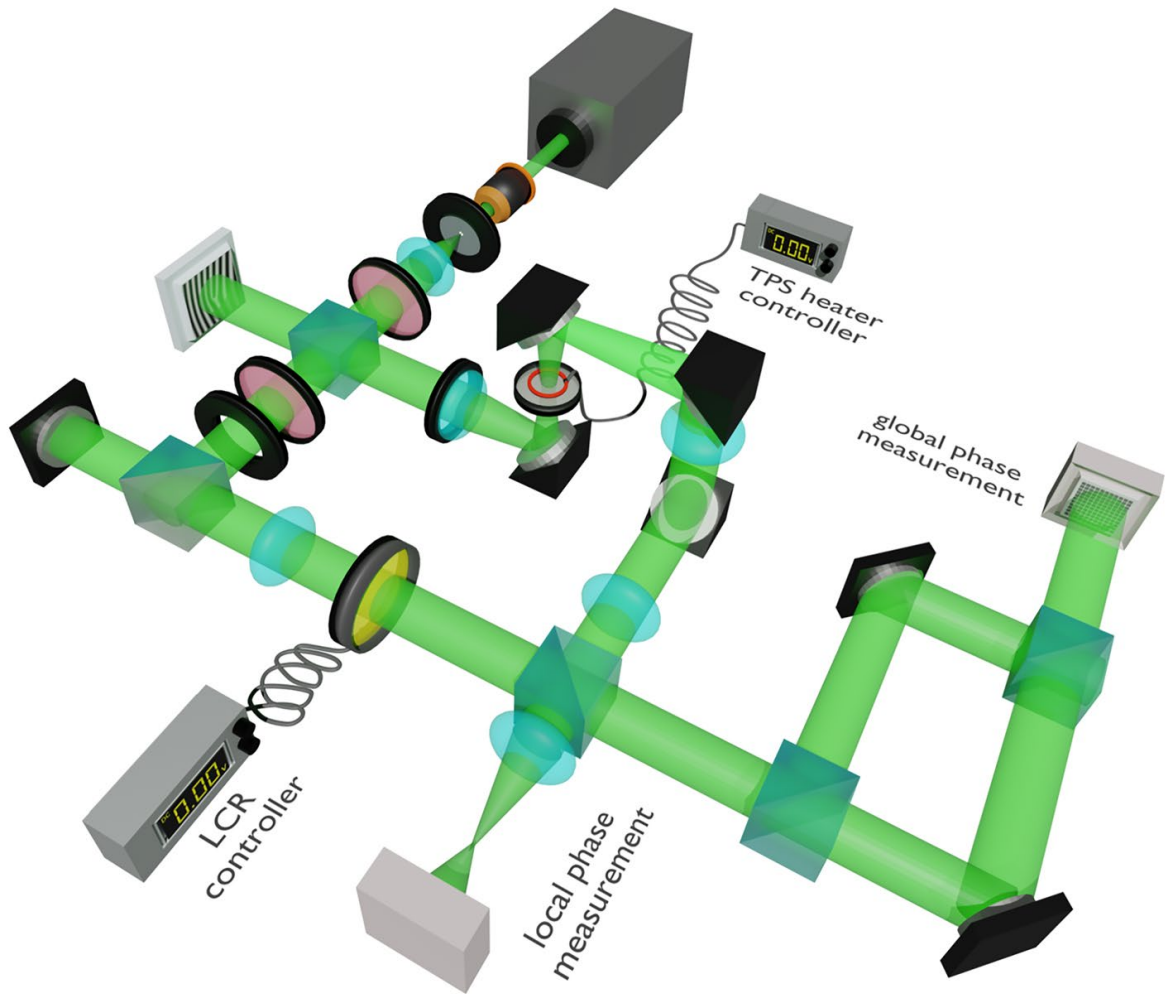
Schematic of experimental setup. The spatially filtered Gaussian beam is divided using a polarization beam splitter. The transmitted beam passes through a lens that generates a spherical wavefront. The second beam reflects off a spatial light modulator (SLM) and then passes through the TPS. Two measurement types are accommodated. The first one, labeled as "global phase measurement", is the self-interference of the scattered beam, which is realized by blocking the reference arm with a mechanical shutter. The second measurement, labeled as "local phase measurement", comprises four separate measurements to infer the spatial distribution of complex scattered field. This is achieved using a liquid crystal retarder (LCR) operated at a rate of hundreds of kHz.

The experimental setup shown in Fig. 3 includes two interferometers; one using a spherical wave as reference and another one that is self-referenced. The laser beam is filtered, collimated, and then divided into two components. One component is scattered off a spatial light modulator (SLM) to generate a Bessel-Gauss beam with variable topological charge $m$. This is subsequently converted into a perfect optical vortex beam (POV) in the back focal plane of an appropriate lens where the TPS is located. The emerging speckle pattern is directed towards a Fourier lens such that a non-evolving speckle field is created^59^. The other component is transformed into a spherical wave that passes through a variable liquid crystal retarder (LCR) and acts as a reference for measuring the phase distribution of the scattered optical field in less than 1 ms. Examples of retrieved phase distributions are illustrated in the Supplementary Materials 3 where we also provide details of the procedure used to infer the topological charge.

For tracking the global phase, we used the self-referenced interferometer by blocking the spherical wave and directing the scattered through a Mach–Zehnder interferometer. The measured intensity distribution evolves towards the typical curved-fork pattern when the strength of randomness decreases as a result of temperature increase in the TPS. Several examples are illustrated in the Supplementary Materials 3.

A number of practical factors affect any experiment. Because the phase assessment relies on intensity measurements, there is an inherent limitation on the spatial regions where the field can be tested. Moreover, in the practice, field is sampled in Cartesian coordinates, which means that an accurate phase gradient can only be determined along a rectangular contour. This complicates a direct comparison with the analytical model but it can be conveniently accounted for in the numerical calculations. To proceed, one must find the range of phase variance which is equivalent to the voltage variation (temperature) in the experiment. Thus, we first determined the statistical properties of the ground glass topography and measured the thermally induced variation in the TPS properties as detailed in the Supplementary Materials 3.

**Figure 4:**
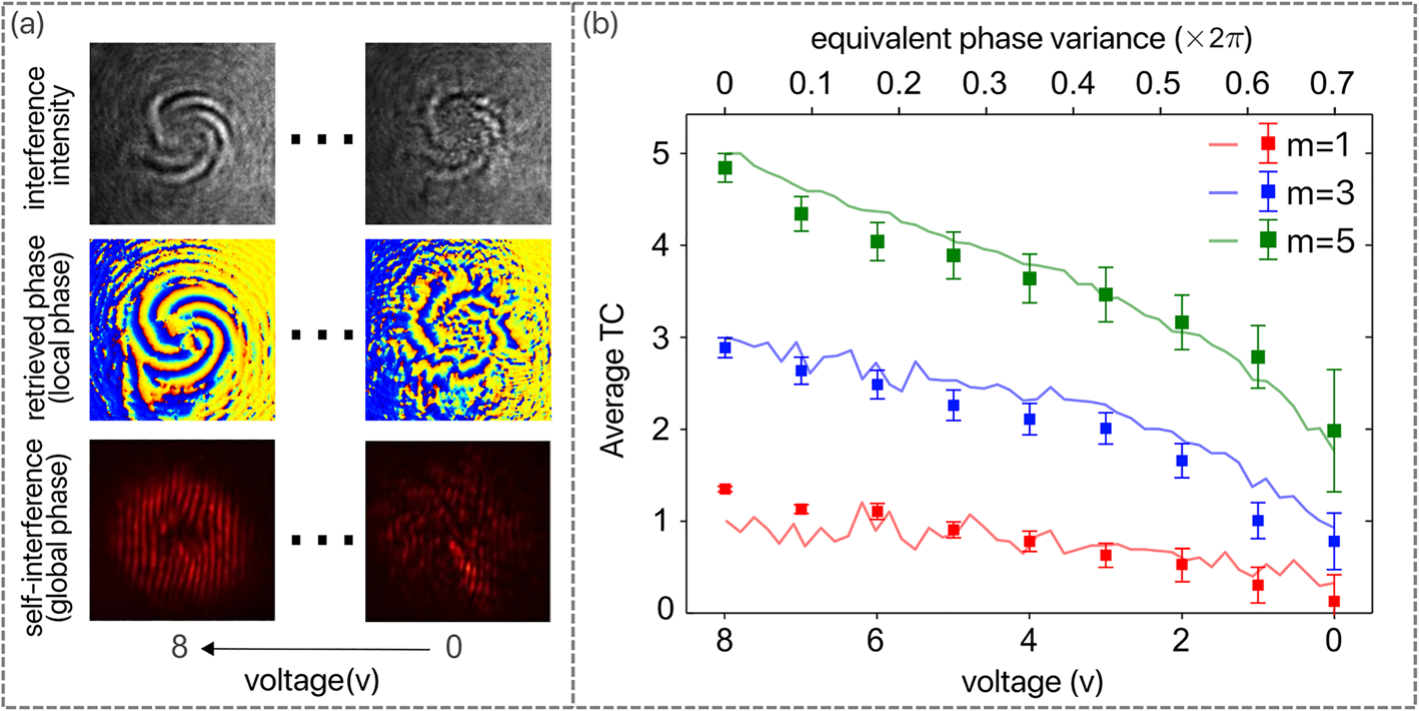
Experimental results. Panel (**a**): an example of measured intensity and phase of the scattered field for case $m = 5$ corresponding to the highest (voltage 0) and the lowest (voltage 8) scattering perturbation. All measurements are for the same propagation distance and the only adjustable parameter is the voltage applied to the heater. Panel (**b**): comparison between the experimental results (solid symbols, bottom axis) and the numerical calculation (solid lines, top axis) conducted for the specific scattering conditions. The experimental results were averaged over four measurements at different locations across the TPS while the numerical data was averaged over 30 realizations of the randomness. For both simulation and experiment, the vorticity was calculated over square contours around the optical axis. The equivalent extent of the phase variance was found based on the measured statistical properties of the TPS.

Examples of recovered phases for lowest and highest voltages of TPS are shown in panel (a) of Fig. 4. In the case of weak randomness (high TPS voltage), the self-interference pattern can be used to estimate the TC because the local vortices emerging from the initial one are still closely spaced such that one single singularity can be resolved. On the other hand, when the randomness increases (low TPS voltage), the emerging local vortices have wandered away from their origin and the field is approaching a homogeneous state. Thus, in order to find the TC, one needs to find instead the local phase distribution. Of course, as opposed to a simple self-interference examination, one now requires a number of different intensity measurements to determine the local phase across the field.

The experimental results for different initial vorticities are summarized in the panel (b) of Fig. 4. Here, the benchmark was a numerical simulation that was conducted for the particular experimental conditions. The numerical simulations permit a direct comparison with the analytical relations derived in polar coordinates to the experimental results evaluated over the rectangular contours afforded by the camera sampling in Cartesian coordinates. Because the numerical simulations can be conducted in both ways, we first confirmed their accuracy by direct comparison with the theoretical expectations, as illustrated in Fig. 1. Then, we simulated the exact conditions of the experiment, evaluating the corresponding topological charge and comparing it with the experimental results, as shown in Fig. 4b. According to these data, the extent of the vortex memory is evident and so is the agreement between the experimental data and the model predictions even though no calibration or adjusting parameters are used in this procedure.

Aside from establishing the extent of vortex memory, our development has a broader impact in the context of characterizing nonuniform fields. A significant consequence of the analytical model is that it establishes a relationship between the average weight of each OAM mode and the vorticity mode characterizing the final random field. In general, there is no deterministic relation between these two properties of inhomogeneous fields, but here, we have derived a clear statistical connection between them as shown in Eq. (7). In other words, using our result, one can extrapolate the information measured along a closed contour to describe both amplitude and phase of the electric field inside the contour. Note that this relationship is independent of the properties of the random media and it depends only on the area over which the measurement is done.

It worth mentioning that similar results are expected when the initial Bessel-Gauss beam is replaced with a Laguerre–Gauss beam. As shown in Refs.^60,61^, POV with similar properties can also be generated using Laguerre–Gauss. Note that there is no connection between the so-called "self-healing" property of these types of beams and the phase memory discussed in this work. Firstly, the notion of self-healing refers to the intensity reconstruction, not the phase distribution. Second, self-healing is a phenomena that happens upon propagation. Here on the other hand, the analysis is done over a constant plane (in the far-field) and the variable is the "strength of the randomness", not the propagation length.

Lastly, the interaction model we developed here can be useful to further study other basic phenomena involving many body interactions, which can be emulated in optical vortex fields. There are current attempts to describe the interaction potentials that govern the vortex-vortex interaction in optical fields but, so far, a clear description is not available.

In closing, we would like to add that our results are directly relevant to the associated stochastic inverse problem. When the characteristics of random media can be appropriately modeled as space-variant phase perturbations, the statistical relations we established between the randomness parameters and the effective vorticity can enable simple sensing strategies where POVs with different vorticities are used to interrogate the randomness. Measurements of global phases in the corresponding inhomogeneous fields could then be used to determine the variance and the spatial correlation of the phase disturbance.

## Supplementary Information

Supplementary Information.

## Data Availability

Data underlying the results presented in this paper are not publicly available at this time but may be obtained from the corresponding author upon reasonable request.
