# Supplementary material for: Phase memory of optical vortex beams
Source: Sci Rep. 2022 Jun 21;12:10428. doi: 10.1038/s41598-022-14074-4 (PMC9213497; doi:10.1038/s41598-022-14074-4)
Supplement: Supplementary file 1 — Supplementary Information. [file 41598_2022_14074_MOESM1_ESM.pdf]

supplementary materials

Phase memory of optical vortex beams

Mahdi Eshaghi[1], Cristian Hernando Acevedo[1], Mahed Batarseh[1], José Rafael Guzman-Sepulveda[1,†], Aristide Dogariu[1,*]

[1] CREOL, The College of Optics and Photonics at the University of Central Florida, 4304 Scorpius St, Orlando, Orlando, Florida 32816, USA.

[†] Currently at the center for Research and Advanced Studies of the National Polytechnic Institute, Apodaca, Nuevo Leon 66600, Mexico.

1. Theory

1.1. Derivation of Eq. (6)

Following Eq. 4 in the main text, we expand every single function described the far-field scattering of the perfect orbital vortex beam of vorticity m, using the Taylor series. Note that the coordinate system is described in Fig. S1. Before proceeding, assume that $A_0 = E_0/N$. One can write

$$
\begin{aligned}
E(\rho,\phi) &= A_0 \sum_{n=1}^{N} e^{i\psi_n}\, e^{im\theta_n}\, e^{-i\mu\rho r_0 \cos(\phi-\theta_n)} \\
&= A_0 \sum_{n=1}^{N} e^{i\psi_n}\, e^{im\theta_n} \sum_{k=0}^{\infty} \frac{(-i\mu\rho r_0)^k}{k!} \left(\frac{e^{i(\phi-\theta_n)} + e^{-i(\phi-\theta_n)}}{2}\right)^k \\
&= A_0 \sum_{n=1}^{N} e^{i\psi_n}\, e^{im\theta_n} \sum_{k=0}^{\infty} \frac{(-i\mu\rho r_0)^k}{2^k\; k!} \sum_{q=0}^{k} \binom{k}{q} \left(e^{i(\phi-\theta_n)}\right)^{k-2q} \\
&= A_0 \sum_{s=0}^{\infty} \sum_{q=0}^{s} \binom{s}{q} \frac{(-i\mu\rho r_0)^s}{2^s\; s!}\, e^{i(s-2q)\phi} \sum_{n=1}^{N} e^{i\psi_n}\, e^{i[m-(s-2q)]\theta_n} \\
&= A_0 \sum_{s=0}^{\infty} \sum_{q=0}^{s} \binom{s}{q} \frac{(-i\mu\rho r_0)^s}{2^s\; s!}\, e^{i(s-2q)\phi} \sum_{n=1}^{N/d} e^{i\psi_n}\, e^{i[m-(s-2q)]\theta_{(n-1)d}} \left[\sum_{p=1}^{d} e^{i[m-(s-2q)]\theta_p}\right] \\
&= A_0 \sum_{s=0}^{\infty} \sum_{q=0}^{s} \binom{s}{q} \frac{(-i\mu\rho r_0)^s}{2^s\; s!}\, e^{i(s-2q)\phi} \sum_{p=1}^{d} e^{i[m-(s-2q)]\theta_p} \sum_{n=1}^{N/d} e^{i\psi_n}\, e^{i[m-(s-2q)]\theta_{(n-1)d}}
\end{aligned}
$$

where d denotes the correlation length of randomness, i.e., the number of units which have the same value of phase. Considering $k = s - 2q$ is a new variable. Since $s > q > 0$ and $\infty > s > 0$, then k can be any integer in the range $[-\infty, \infty]$. For specific value of k, $q = (s-k)/2$. One can write

$$
\begin{aligned}
E(\rho,\phi) &= A_0 \sum_{s=0}^{\infty} \sum_{q=0}^{s} \binom{s}{q} \frac{(-i\mu\rho r_0)^s}{2^s\; s!}\, e^{i(s-2q)\phi} \sum_{p=1}^{d} e^{i[m-(s-2q)]\theta_p} \sum_{n=1}^{N/d} e^{i\psi_n}\, e^{i[m-(s-2q)]\theta_{(n-1)d}} \\
&= A_0 \sum_{k=-\infty}^{0} \sum_{s=-k}^{\infty} \binom{s}{(s-k)/2} \frac{(-i\mu\rho r_0)^s}{2^s\; s!}\, e^{ik\phi} \sum_{p=1}^{d} e^{i[m-k]\theta_p} \sum_{n=1}^{N/d} e^{i\psi_n}\, e^{i[m-k]\theta_{(n-1)d}} \\
&\quad + A_0 \sum_{k=0}^{\infty} \sum_{s=k}^{\infty} \binom{s}{(s-k)/2} \frac{(-i\mu\rho r_0)^s}{2^s\; s!}\, e^{ik\phi} \sum_{p=1}^{d} e^{i[m-k]\theta_p} \sum_{n=1}^{N/d} e^{i\psi_n}\, e^{i[m-k]\theta_{(n-1)d}} \\
&= A_0 \sum_{k=-\infty}^{\infty} \left[\sum_{s=0}^{\infty} \binom{2s+|k|}{s} \frac{(-i\mu\rho r_0)^{2s+|k|}}{2^{2s+|k|}(2s+|k|)!} \sum_{p=1}^{d} e^{i[m-k]\theta_p} \sum_{n=1}^{N/d} e^{i\psi_n}\, e^{i[m-k]\theta_{(n-1)d}}\right] e^{ik\phi} \\
&= A_0 \sum_{k=-\infty}^{\infty} H(k;\rho)\, e^{ik\phi}
\end{aligned}
$$

where

$$
H(k;\rho) = A_0 \sum_{s=0}^{\infty} \binom{2s+|k|}{s} \frac{(-i\mu\rho r_0)^{2s+|k|}}{2^{2s+|k|}(2s+|k|)!} \sum_{p=1}^{d} e^{i[m-k]\theta_p} \sum_{n=1}^{N/d} e^{i\psi_n}\, e^{i[m-k]\theta_{(n-1)d}}\,.
$$

As one can recognize, these three summations are independent of each other. The first part can be written as

$$
\sum_{s=0}^{\infty} \binom{2s+|k|}{s} \frac{(-i)^{2s+|k|}\;\beta^{2s+|k|}}{2^{2s+|k|}\;(2s+|k|)!} = \sum_{s=0}^{\infty} \frac{(-i)^{2s+|k|}\;\beta^{2s+|k|}}{2^{2s+|k|}\;s!\;(s+|k|)!}
$$

$$
\begin{aligned}
&= (-i)^{|k|} \sum_{s=0}^{\infty} \frac{(-1)^s}{s!\,(s+|k|)!} \left(\frac{\beta}{2}\right)^{2s+|k|} \\
&= (-i)^{|k|}\ \mathrm{J}_{|k|}(\beta)\ .
\end{aligned}
$$

We can also write the equivalent relation for the second part of the coefficient as

$$
\begin{aligned}
\sum_{p=1}^{d} e^{i[m-k]\theta_p} &= \sum_{p=1}^{d} e^{ip[m-k]\left(\frac{2\pi}{N}\right)} \\
&= \frac{\sin\left([m-k]\frac{d}{N}\pi\right)}{\sin\left([m-k]\frac{1}{N}\pi\right)}\ e^{i[m-k]\,\frac{d+1}{N}\,\pi} \\
&\approx d\ \mathrm{sinc}\left([m-k]\frac{d}{N}\pi\right) e^{i[m-k]\,\frac{d+1}{N}\,\pi}
\end{aligned}
$$

where the condition $d \ll N$ was considered. Replacing the first two terms of the coefficient, $H(k;\rho)$ will be

$$
H(k;\rho) = \left[(-i)^{|k|} e^{i[m-k]\,\frac{d+1}{N}\,\pi}\right] \left[A_0\ \mathrm{J}_{|k|}(\beta)\ d\,\mathrm{sinc}\left([m-k]\frac{d}{N}\pi\right)\right] \left[\sum_{n=1}^{N/d} e^{i\psi_n}.\, e^{i[m-k]\theta_{(n-1)d}}\right].
$$

Because we want to continue in the general case, we assume that randomness has a probability distribution $p(\psi')$ over the mean phase ψ_0. This way, the meaning of ψ' will be *the phase disturbance.* Therefore, one can rewrite the above equation as

$$
H(k;\rho) = \left[(-i)^{|k|} e^{i[m-k]\,\frac{d+1}{N}\,\pi} e^{i\psi_0}\right] \left[A_0\ \mathrm{J}_{|k|}(\beta)\ d\,\mathrm{sinc}\left([m-k]\frac{d}{N}\pi\right)\right] \left[\sum_{n=1}^{N/d} e^{i\psi'_n}.\, e^{i[m-k]\theta_{(n-1)d}}\right].
$$

For proceeding into the next step, we define $P = (-i)^{|k|} e^{i[m-k]\,\frac{d+1}{N}\,\pi} e^{i\psi_0}$ as the *constant phase* and $A = A_0\ \mathrm{J}_{|k|}(\beta)\ d\,\mathrm{sinc}([m-k]\frac{d}{N}\pi)$ as sort of an *amplitude* term, except the last stochastic summation where we have

$$
H(k;\rho) = P.\,A\ \sum_{n=1}^{N/d} e^{i\psi'_n}.\, e^{i[m-k]\theta_{(n-1)d}}\ .
$$

In case of no randomness, we have

$$
\begin{aligned}
H(k;\rho) &= P.\,A\ \sum_{n=1}^{N/d} e^{i[m-k]\theta_{(n-1)d}} \\
&= P.\,A(N/d)\ \delta(m-k) \\
&= NA_0\ (-i)^{|k|}\ \mathrm{J}_{|m|}(\mu\rho r_0)
\end{aligned}
$$

where $NA_0 = E_0$ and therefore

$$
\begin{aligned}
&H(k;\rho) = E_0\ (-i)^{|k|}\ \mathrm{J}_{|m|}(\mu\rho r_0) \\
&H(k \neq m;\rho) = 0.
\end{aligned}
$$

Finally, we have

$$
E(\rho,\phi) = E_0\ (-i)^{|k|}\ \mathrm{J}_{|m|}(\mu\rho r_0)\ e^{im\phi}.
$$

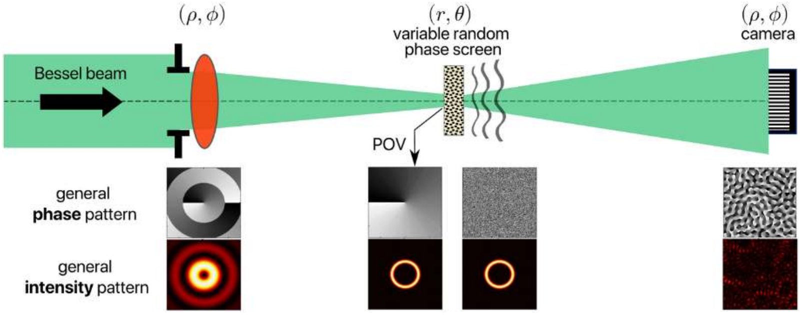

Fig. S1. Conceptual implementation. Fourier transform of a Bessel beam results in "perfect optical vortex" beam (POV). The random field caused by POV scattered by random phase screen in the far-field regime is considered in this work.

1.2. Statistical average of vorticity and OAM modes

To derive the probability density for $H(k)$ and thus find the statistical properties of the random field, we first need to separate real and imaginary parts, as

$$
\begin{aligned}
\mathrm{Re}\{H(k;\rho)\} = A\sum_{n=1}^{N/d} & \cos(\psi_n')\left\{\mathrm{Re}\{P\}\cos([m-k]\theta_{(n-1)d}) - \mathrm{Im}\{P\}\sin([m-k]\theta_{(n-1)d})\right\} \\
& - A \sum_{n=1}^{N/d} \sin(\psi_n')\left\{\mathrm{Re}\{P\}\sin([m-k]\theta_{(n-1)d}) + \mathrm{Im}\{P\}\cos([m-k]\theta_{(n-1)d})\right\} \\
\mathrm{Im}\{H(k;\rho)\} = A\sum_{n=1}^{N/d} & \cos(\psi_n')\left\{\mathrm{Im}\{P\}\cos([m-k]\theta_{(n-1)d}) + \mathrm{Re}\{P\}\sin([m-k]\theta_{(n-1)d})\right\} \\
& + A\sum_{n=1}^{N/d} \sin(\psi_n')\left\{-\mathrm{Im}\{P\}\sin([m-k]\theta_{(n-1)d}) + \mathrm{Re}\{P\}\cos([m-k]\theta_{(n-1)d})\right\}.
\end{aligned}
$$

One then needs to find the average and variance of both terms $\sin(\psi_n')$ and $\cos(\psi_n')$. Taking advantage of the *normalized Gaussian probability distribution,* we have

$$
p(\psi') = \begin{cases} \left(\sqrt{2\pi s^2}\ \mathrm{erf}(\alpha\pi/\sqrt{2s^2})\right)^{-1} e^{-\frac{1}{2}\left(\frac{\psi'^2}{s^2}\right)} & -\alpha\pi < \psi' < \alpha\pi \\ 0 & \textit{else}\ . \end{cases}
$$

Thus, we can calculate the desired averages and variances (see below table).

$\langle\cos(\psi_n')\rangle = C_1 = e^{-s^2/2}\left[\dfrac{\mathrm{erf}\left(\frac{\alpha\pi - is^2}{\sqrt{2}s}\right) + \mathrm{erf}\left(\frac{\alpha\pi + is^2}{\sqrt{2}s}\right)}{2\ \mathrm{erf}\left(\frac{\alpha\pi}{\sqrt{2}s}\right)}\right]$		
$\langle\cos^2(\psi_n')\rangle = C_2 = e^{-s^2/2}\left[\dfrac{2\ e^{2s^2}\ \mathrm{erf}\left(\frac{\alpha\pi}{\sqrt{2}s}\right) + \mathrm{erf}\left(\frac{\alpha\pi - 2is^2}{\sqrt{2}s}\right) + \mathrm{erf}\left(\frac{\alpha\pi + 2is^2}{\sqrt{2}s}\right)}{4\ \mathrm{erf}\left(\frac{\alpha\pi}{\sqrt{2}s}\right)}\right]$		
$\langle\sin^2(\psi_n')\rangle = 1 - \langle\cos^2(\psi_n')\rangle = 1 - C_2$	$\langle\cos(\psi_n')\sin(\psi_n')\rangle = 0$	$\langle\sin(\psi_n')\rangle = 0$

Before proceeding, we need to remind that $P = (-i)^{|k|}e^{i[m-k]\frac{d+1}{N}\pi}e^{i\psi_0}$ where ψ_0 determines the *average phase* introduced by the randomness (center of the Gaussian distribution). Thus, $P = e^{i\varphi_P}$ is a pure phase, and we can use the argument φ_P in the below calculations. Using the values we found in the above table, average and variance for both real and imaginary parts of $H(k)$ can be calculated,

$$
\begin{aligned}
\langle\mathrm{Re}\{H(k;\rho)\}\rangle &= A\ C_1\sum_{n=1}^{N/d}\{\mathrm{Re}\{P\}\cos([m-k]\theta_{(n-1)d}) - \mathrm{Im}\{P\}\sin([m-k]\theta_{(n-1)d})\} \\
&= (N/d)A\ C_1\ \mathrm{Re}\{P\}\delta(k-m) \\
\langle\mathrm{Im}\{H(k;\rho)\}\rangle &= A\ C_1\sum_{n=1}^{N/d}\{\mathrm{Re}\{P\}\cos([m-k]\theta_{(n-1)d}) - \mathrm{Im}\{P\}\sin([m-k]\theta_{(n-1)d})\} \\
&= (N/d)A\ C_1\ Im\{P\}\delta(k-m) \\
\langle\mathrm{Re}^2\{H(k;\rho)\}\rangle &= A^2\ \sum_{n=1}^{N/d}\{\mathrm{Re}\{P\}\ \cos([m-k]\theta_{(n-1)d}) - \mathrm{Im}\{P\}\sin([m-k]\theta_{(n-1)d})\}^2 \\
&\quad + A^2\ \langle\cos(\psi_n')\rangle^2\left[\sum_{n=1}^{N/d}\mathrm{Re}\{P\}\ \cos([m-k]\theta_{(n-1)d}) - \mathrm{Im}\{P\}\sin([m-k]\theta_{(n-1)d})\right]^2 \\
&\quad + A^2\ \langle\sin^2(\psi_n')\rangle\ \sum_{n=1}^{N/d}\{\mathrm{Re}\{P\}\sin([m-k]\theta_{(n-1)d}) + \mathrm{Im}\{P\}\cos([m-k]\theta_{(n-1)d})\}^2 \\
&= (N/d)(A^2/2)\{[1-C_1^2] + [2C_2 - C_1^2 - 1]\ \cos(2\varphi_P)\delta(k-m) \\
&\quad + (N/d)[2C_1^2]\ \cos^2(\varphi_P)\delta(m-k)\} \\
\langle\mathrm{Im}^2\{H(k;\rho)\}\rangle &= A^2\ \sum_{n=1}^{N/d}\{\mathrm{Im}\{P\}\ \cos([m-k]\theta_{(n-1)d}) + \mathrm{Re}\{P\}\sin([m-k]\theta_{(n-1)d})\}^2 \\
&\quad + A^2\ \langle\cos(\psi_n')\rangle^2\left[\sum_{n=1}^{N/d}\mathrm{Im}\{P\}\ \cos([m-k]\theta_{(n-1)d}) + \mathrm{Re}\{P\}\sin([m-k]\theta_{(n-1)d})\right]^2 \\
&\quad + A^2\ \langle\sin^2(\psi_n')\rangle\ \sum_{n=1}^{N/d}\{-\mathrm{Im}\{P\}\sin([m-k]\theta_{(n-1)d}) + \mathrm{Re}\{P\}\cos([m-k]\theta_{(n-1)d})\}^2 \\
&= (N/d)(A^2/2)\{[1-C_1^2] - [2C_2 - C_1^2 - 1]\ \cos(2\varphi_P)\delta(k-m) \\
&\quad + (N/d)[2C_1^2]\ \sin^2(\varphi_P)\delta(m-k)\}
\end{aligned}
$$

$$
\begin{aligned}
&\langle\mathrm{Re}\{H(k;\rho)\}\mathrm{Im}\{H(k;\rho)\}\rangle \\
&\quad = (N/d)(A^2/2)\{[2C_2 - C_1^2 - 1]\ \sin(2\varphi_P)\delta(k-m) + (N/d)\ [C_1^2]\ \sin(2\varphi_P)\delta(m-k)\}.
\end{aligned}
$$

The correlation coefficient $g_{Re,Im}$ which is defined as normalized covariance of real and imaginary parts can be found as $g_{\mathrm{Re,Im}} = \langle(\mathrm{Re}-\overline{\mathrm{Re}})(\mathrm{Im}-\overline{\mathrm{Im}})\rangle/\sigma_{\mathrm{Re}}\sigma_{\mathrm{Im}} = [\overline{\mathrm{Re}\ \mathrm{Im}} - \overline{\mathrm{Re}}\ \overline{\mathrm{Im}}]/\sigma_{\mathrm{Re}}\sigma_{\mathrm{Im}}$ where $\sigma_{\mathrm{Re}} = \sqrt{\overline{\mathrm{Re}^2} - \overline{\mathrm{Re}}^2}$ and $\sigma_{\mathrm{Im}} = \sqrt{\overline{\mathrm{Im}^2} - \overline{\mathrm{Im}}^2}$ are standard deviations of real and imaginary parts, respectively. Substituting the above calculated values, all required parameters will be achieved.

$\overline{\mathrm{Re}} = (N/d)A\ C_1\ \cos(\varphi_P)\ \delta(k-m)$	$\overline{\mathrm{Im}} = (N/d)A\ C_1\ \sin(\varphi_P)\ \delta(k-m)$
$\sigma^2_{\mathrm{Re}} = (N/d)(A^2/2)\{[1-C_1^2] + [2C_2 - C_1^2 - 1]\ \cos(2\varphi_P)\ \delta(k-m)\}$	
$\sigma^2_{\mathrm{Im}} = (N/d)(A^2/2)\{[1-C_1^2] - [2C_2 - C_1^2 - 1]\ \cos(2\varphi_P)\ \delta(k-m)\}$	
$g_{\mathrm{Re,Im}}\sigma_{\mathrm{Re}}\sigma_{\mathrm{Im}} = (N/d)\ (A^2/2)\ [2C_2 - C_1^2 - 1]\ \sin(2\varphi_P)\ \delta(k-m)$	

When the number of random complex components inside a summation is large enough, one can take advantage of *Central Limit Theorem,* according to which the joint density function of Re and Im has a general Gaussian distribution, as below

$$p_{\mathrm{Re,Im}} = \frac{1}{2\pi\sigma_{\mathrm{Re}}\sigma_{\mathrm{Im}}\sqrt{1-g^2_{\mathrm{Re,Im}}}} .\ exp\left[-\frac{\left(\frac{\mathrm{Re}-\overline{\mathrm{Re}}}{\sigma_{\mathrm{Re}}}\right)^2 + \left(\frac{\mathrm{Im}-\overline{\mathrm{Im}}}{\sigma_{\mathrm{Im}}}\right)^2 - 2g_{\mathrm{Re,Im}}\left(\frac{\mathrm{Re}-\overline{\mathrm{Re}}}{\sigma_{\mathrm{Re}}}\right)\left(\frac{\mathrm{Im}-\overline{\mathrm{Im}}}{\sigma_{\mathrm{Im}}}\right)}{2(1-g^2_{\mathrm{Re,}\ \ })}\right].$$

One can find the probability distribution for amplitude and phase $(\tilde{A},\tilde{\theta})$ of this complex parameter taking advantage of the Jacobian transformation $p_{\tilde{A},\tilde{\theta}}(\tilde{A},\tilde{\theta}) = p_{\mathrm{Re,Im}}(\tilde{A}\cos(\theta), \tilde{A}\sin(\theta)) \times \|J\|$ where $\|J\|$ is the determinant of the Jacobian matrix, which equals to $\tilde{A}$ in this case. Thus, we have

$$p_{\tilde{A},\tilde{\theta}} = \frac{\tilde{A}}{2\pi\sigma_{\mathrm{Re}}\sigma_{\mathrm{Im}}\sqrt{1-g^2_{\mathrm{Re,Im}}}} exp\left[-\frac{\left(\frac{\tilde{A}\cos(\tilde{\theta})-\overline{\mathrm{Re}}}{\sigma_{\mathrm{Re}}}\right)^2 + \left(\frac{\tilde{A}\sin(\tilde{\theta})-\overline{\mathrm{Im}}}{\sigma_{\mathrm{Im}}}\right)^2 - 2g_{\mathrm{Re,Im}}\left(\frac{\tilde{A}\cos(\tilde{\theta})-\overline{\mathrm{Re}}}{\sigma_{\mathrm{Re}}}\right)\left(\frac{\tilde{A}\sin(\tilde{\theta})-\overline{\mathrm{Im}}}{\sigma_{\mathrm{Im}}}\right)}{2(1-g^2_{\mathrm{Re,Im}})}\right].$$

Let us consider two general conditions which can exist and find the probability distribution regarding each one. We will use the expressions we found in the above table.

Condition $1{:}\,k \neq m$

$\overline{\mathrm{Re}} = 0$	$\overline{\mathrm{Im}} = 0$
$\sigma^2_{\mathrm{Re}} = (N/d)(A^2/2)\ [1-C_1^2]$	
$\sigma^2_{\mathrm{Im}} = (N/d)(A^2/2)\ [1-C_1^2]$	
$g_{\mathrm{Re,Im}}\sigma_{\mathrm{Re}}\sigma_{\mathrm{Im}} = 0$	

For this case, the probability distribution will be as below ($\sigma_{\mathrm{Re}} = \sigma_{\mathrm{Im}} = \sigma$)

$$p_{\tilde{A},\tilde{\theta}} = \frac{\tilde{A}}{2\pi\sigma^2}\exp\left[-\frac{\tilde{A}^2}{2\sigma^2}\right] \ \rightarrow\ p_{\tilde{A}} = \int_{-\pi}^{\pi} p_{\tilde{A},\tilde{\theta}}\ d\tilde{\theta} \ \rightarrow\ p_{\tilde{A}} = \frac{\tilde{A}}{\sigma^2}\exp\left[-\frac{\tilde{A}^2}{2\sigma^2}\right]$$

$$\rightarrow\ p^{k\neq m}_{|H(k)|} = \frac{|H(k)|}{(N/d)(A^2/2)\ [1-C_1^2]}\ \exp\left[-\frac{|H(k)|^2}{(N/d)(A^2/2)\ [1-C_1^2]}\right].$$

As it was mentioned in the main text, $A = A_0 \mathrm{J}_{|k|}(\beta)\ d\,\mathrm{sinc}([m-k](d/N)\pi)$, where $\beta = \mu\rho r_0$ and $\mu = \pi/\lambda z$. By replacing parameter A in the above relation, we have

$$\boxed{p^{k\neq m}_{|H(k;\rho)|} = \frac{|H(k;\rho)|}{(Nd/2)[1-C_1^2]\ [\mathrm{J}_{|k|}(\beta)\,\mathrm{sinc}([m-k](d/N)\pi)]^2}\ exp\left[-\frac{|H(k;\rho)|^2}{(Nd)[1-C_1^2]\ [\mathrm{J}_{|k|}(\beta)\,\mathrm{sinc}([m-k](d/N)\pi)]^2}\right]}$$

Condition 2: $k = m$

$\overline{\mathrm{Re}} = (N/d)A\ C_1\ \cos(\varphi_P)$	$\overline{\mathrm{Im}} = (N/d)A\ C_1\ \sin(\varphi_P)$
$\sigma_{\mathrm{Re}}^2 = (N/d)(A^2/2)\{[1 - C_1^2] + [2C_2 - C_1^2 - 1]\ \cos(2\varphi_P)\}$	
$\sigma_{\mathrm{Im}}^2 = (N/d)(A^2/2)\{[1 - C_1^2] - [2C_2 - C_1^2 - 1]\ \cos(2\varphi_P)\}$	
$g_{\mathrm{Re,Im}}\sigma_{\mathrm{Re}}\sigma_{\mathrm{Im}} = (N/d)(A^2/2)\ [2C_2 - C_1^2 - 1]\ \sin(2\varphi_P)$	

The case of $k = m$ is more complicated than the previews case because the standard deviations and averages are different for real and imaginary parts. That is the reason that we simplify the relation into below one

$$p_{|H(k;\rho)|}^{k=m} = \frac{|H(k;\rho)|}{2\pi A^2\sqrt{t}}\ e^{-t_1}\ e^{-(t_2/A^2)|H(k;\rho)|^2} \int_{-\pi}^{\pi} \exp\Big[(t_3/A^2)|H(k;\rho)|^2\ \cos(2(\tilde{\theta} - \varphi_P)) + (t_4/A)|H(k;\rho)|\ \cos(\tilde{\theta} - \varphi_P)\Big]\ d\tilde{\theta}$$

where parameters t, t_1, t_2, t_3, and t_4 are all *non-negative* and defined as

$$\begin{aligned} t &= (N/d)^2[1 - C_2][C_2 - C_1^2] \\ t_1 &= \frac{1}{2}(N/d)\left[\frac{C_1^2}{C_2 - C_1^2}\right] \\ t_2 &= \frac{1}{4}\frac{1}{(N/d)}\left[\frac{[1 - C_1^2]}{[1 - C_2][C_2 - C_1^2]}\right] \\ t_3 &= \frac{1}{4}\frac{1}{(N/d)}\left[\frac{[2C_2 - C_1^2 - 1]}{[1 - C_2][C_2 - C_1^2]}\right] \\ t_4 &= \left[\frac{C_1}{C_2 - C_1^2}\right], \end{aligned}$$

and $A = A_0 \mathrm{J}_{|k|}(\beta)\ d\,\mathrm{sinc}([m - m](d/N)\pi) = A_0\ \mathrm{J}_{|k|}(\beta)\ d$. Note that no parameter is function of phase φ_P. Therefore, we find that the phase φ_P or any term inside that, especially ψ_0, *does not* affect the probability distributions at all, neither the case $k \neq m$ nor $k = m$. One can take advantage of below integral

$$\int_{-\pi}^{\pi} \exp\big[z\ \cos(\ell\tilde{\theta}) + y\ \cos(\tilde{\theta})\big]\ d\tilde{\theta} = 2\pi\ [\mathrm{I}_0(\pm z)\mathrm{I}_0(-y) + 2\sum_{n=1}^{\infty} \mathrm{I}_n(\pm z)\mathrm{I}_{|m|n}(-y)]$$

where $\pm$ belong to even (positive) or odd (negative) value of ℓ, and I_n shows the modified Bessel function of order n. Substituting $\ell = 2$, we have

$$\boxed{p_{|H(k;\rho)|}^{k=m} = \frac{|H(k;\rho)|}{A^2\sqrt{t}}\ e^{-t_1}\ e^{-A^2 t_2\ |H(k;\rho)|^2} \Big\{\mathrm{I}_0(A^2 t_3 |H(k;\rho)|^2)\ \mathrm{I}_0(-A t_4 |H(k;\rho)|) + 2\sum_{n=1}^{\infty} \mathrm{I}_n(A^2 t_3 |H(k;\rho)|^2)\ \mathrm{I}_{2n}(-A t_4 |H(k;\rho)|)\Big\}}$$

Now that we have found the probability density functions, we can move toward the next step. As it was mentioned in the main paper, we want to calculate the weight of each *Vorticity* (V^k) and *OAM* (L^k) modes of order k, inside a circular contour of the radius of ρ_0, using below relations

$$V^k = \langle |H(k;\rho)|^2 \rangle |_{\rho=\rho_0}$$

$$L^k = \int_0^{\rho_0} \langle |H(k;\rho)|^2 \rangle\ \rho\, d\rho\,.$$

Following this, one can find the most probable detected vorticity and OAM mode using [1,2]

$$\langle V \rangle = \frac{\sum_{k=-\infty}^{\infty} k\ V^k}{\sum_{k=-\infty}^{\infty} V^k}$$

$$\langle L \rangle = \frac{\sum_{k=-\infty}^{\infty} k\ L^k}{\sum_{k=-\infty}^{\infty} L^k}\,.$$

Therefore, we need to find the $\langle |H(k;\rho)|^2 \rangle$ using the probability distributions which have been found for both conditions. For the case of $k \neq m$, one can easily show that

$$\langle |H(k;\rho)|^2 \rangle = \int_0^{\infty} |H(k;\rho)|^2\ p_{|H(k)|}^{k\neq m}\ d|H(k;\rho)|\ = (Nd)[1 - C_1^2]\ [\mathrm{J}_{|k|}(\beta)\,\mathrm{sinc}([m - k](d/N)\pi)]^2.$$

In the case of $k = m$, the calculation will be a bit complicated. We will show the steps below, and we use the valuable integrals in each step.

$$\langle |H(k;\rho)|^2\rangle = \int_0^\infty |H(k;\rho)|^2 \; p_{|H(k;\rho)|}^{k=m} \;\; d|H(k;\rho)|$$

$$= \int_0^\infty |H(k;\rho)|^2 \; \frac{|H(k;\rho)|}{2\pi A^2\sqrt{t}} \; e^{-t_1} \; e^{-A^2 t_2\,|H(k;\rho)|^2} \; d|H(k;\rho)| \left[\int_{-\pi}^{\pi} e^{A^2 t_3 |H(k;\rho)|^2 \cos\left(2\tilde{\theta}\right) + A t_4 |H(k;\rho)| \cos\left(\tilde{\theta}\right)} \; d\tilde{\theta}\right]$$

$$= \frac{e^{-t_1}}{2\pi A^2\sqrt{t}} \int_0^\infty |H(k;\rho)|^3 \; e^{-A^2 t_2\,|H(k;\rho)|^2} \; d|H(k;\rho)| \; \int_{-\pi}^{\pi} e^{A^2 t_3 |H(k;\rho)|^2 \cos\left(2\tilde{\theta}\right) + A t_4 |H(k;\rho)| \cos\left(\tilde{\theta}\right)} \; d\tilde{\theta}$$

$$= \frac{e^{-t_1}}{2\pi A^2\sqrt{t}} \int_{-\pi}^{\pi} d\tilde{\theta} \; \int_0^\infty |H(k;\rho)|^3 \; e^{-A^2\left(t_2 - t_3\cos\left(2\tilde{\theta}\right)\right)|H(k;\rho)|^2 + A\left(t_4\cos\left(\tilde{\theta}\right)\right)|H(k;\rho)|} \; d|H(k;\rho)|$$

Replacing $a = A^2\left(t_2 - t_3\cos\left(2\tilde{\theta}\right)\right)$ and $b = A\left(t_4\cos\left(\tilde{\theta}\right)\right)$, we have

$$\langle |H(k;\rho)|^2\rangle = \frac{e^{-t_1}}{2\pi A^2\sqrt{t}} \int_{-\pi}^{\pi} d\tilde{\theta} \; \int_0^\infty |H(k;\rho)|^3 \; e^{-A^2\left(t_2 - t_3\cos\left(2\tilde{\theta}\right)\right)|H(k;\rho)|^2 + A\left(t_4\cos\left(\tilde{\theta}\right)\right)|H(k;\rho)|} \; d|H(k;\rho)|$$

$$= \frac{e^{-t_1}}{2\pi A^2\sqrt{t}} \int_{-\pi}^{\pi} \; \left\{\frac{1}{2}a^{-2} + \frac{1}{8}a^{-3}b^2\right\} + \frac{1}{16}\left\{6a^{-5/2}b + a^{-7/2}b^3\right\} \;\; exp\left(\frac{b^2}{4a}\right)\sqrt{\pi}\;\left[1 + \mathrm{erf}\left(\frac{b}{2\sqrt{a}}\right)\right] \; d\tilde{\theta}$$

$$= \frac{e^{-t_1}}{2\pi A^2\sqrt{t}} \int_{-\pi}^{\pi} \; \left\{\frac{1}{2}a^{-2} + \frac{1}{8}a^{-3}b^2\right\} d\tilde{\theta}$$

$$+ \frac{e^{-t_1}}{2\pi A^2\sqrt{t}} \int_{-\pi}^{\pi} \frac{1}{16}\left\{6a^{-5/2}b + a^{-7/2}b^3\right\} \; exp\left(\frac{b^2}{4a}\right)\sqrt{\pi}\;\left[1 + \mathrm{erf}\left(\frac{b}{2\sqrt{a}}\right)\right] \; d\tilde{\theta}$$

Changing the integration variable from $\tilde{\theta}$ to $u = b/2\sqrt{a}$ for the second integral, we will have

$$\langle |H(k;\rho)|^2\rangle = \frac{e^{-t_1}}{2\pi\sqrt{t}}A^2 \int_{-\pi}^{\pi} \frac{1}{2(t_2 - t_3\;\cos(2\tilde{\theta}))^2} \; d\tilde{\theta} + \frac{e^{-t_1}}{2\pi\sqrt{t}}A^2 \int_{-\pi}^{\pi} \frac{(t_4\;\cos(\tilde{\theta}))^2}{8(t_2 - t_3\;\cos(2\tilde{\theta}))^3} \; d\tilde{\theta}$$

$$+ \frac{e^{-t_1}}{2\pi\sqrt{t}}A^2 \; 4h \int_0^{1/\epsilon} \mathrm{erf}(u)\, e^{u^2} \; (1 - \epsilon^2 u^2)^{-1/2} \; \{h_1 u + h_3 u^3 + h_5 u^5\} \; du$$

where coefficients u_0, h, h_1, h_3, and h_5 are

$$h_1 = \frac{3t_4}{2}, \quad h_3 = t_4 + \frac{12t_3}{t_4}, \quad h_5 = \frac{8t_3}{t_4}, \quad h = \frac{\sqrt{\pi}}{(t_2 + t_3)^{3/2}\;t_4^2}, \qquad \epsilon = 4\left(\frac{t_2 - t_3}{t_4^2}\right).$$

Finally, one can complete the calculations by defining below functions,

$$M_1(t_2, t_3) \equiv \int_{-\pi}^{\pi} \frac{1}{2(t_2 - t_3\;\cos(2\tilde{\theta}))^2} \; d\tilde{\theta} = \frac{t_2\;\pi}{(t_2^2 - t_3^2)^{3/2}}$$

$$M_2(t_2, t_3, t_4) \equiv \int_{-\pi}^{\pi} \frac{(t_4\;\cos(\tilde{\theta}))^2}{8(t_2 - t_3\;\cos(2\tilde{\theta}))^3} \; d\tilde{\theta} = \frac{(2t_2 + t_3)t_4^2\;\pi}{16(t_2 - t_3)^{5/2}\;(t_2 + t_3)^{3/2}}$$

$$F_1(\epsilon) \equiv \int_0^{1/\epsilon} \mathrm{erf}(u) \; e^{u^2} \; (1 - \epsilon^2 u^2)^{-1/2} \; u \; du = \left[\epsilon^{-1}\; e^{1/\epsilon^2}\; \gamma\left(1, \frac{1}{\epsilon^2}\right)\right]$$

$$F_3(\epsilon) \equiv \int_0^{1/\epsilon} \mathrm{erf}(u) \;\; e^{u^2} \; (1 - \epsilon^2 u^2)^{-1/2} \; u^3 \; du = \frac{1}{3}\left[2\epsilon^{-5} - e^{1/\epsilon^2}\;(\epsilon^{-1} - 2\epsilon^{-3})\;\gamma\left(2, \frac{1}{\epsilon^2}\right)\right]$$

$$F_5(\epsilon) \equiv \int_0^{1/\epsilon} \mathrm{erf}(u)\, e^{u^2} \; (1 - \epsilon^2 u^2)^{-1/2} \; u^5 \; du = \frac{2}{15}\left[\epsilon^{-9}(\epsilon^2 + 2) + \epsilon^{-5}\; e^{1/\epsilon^2}\;(2 - 3\epsilon^2 + 3\epsilon^4)\;\gamma\left(3, \frac{1}{\epsilon^2}\right)\right]$$

where γ is the *lower incomplete gamma function*. We have reached the expression for $\langle |H(k;\rho)|^2\rangle$ in the case of $k = m$, as below

$$\langle |H(k;\rho)|^2\rangle = \frac{e^{-t_1}}{2\pi\sqrt{t}}A^2\{M_1(t_2, t_3) + M_2(t_2, t_3, t_4) + 4h\;[h_1 F_1(\epsilon) + h_3 F_3(\epsilon) + h_5 F_5(\epsilon)]\}.$$

For measuring vorticity orders V^k, we just need to replace $\rho = \rho_0$, equivalently, $\beta = \mu\rho_0 r_0$. However, to find L^k, one needs to operate one more integration over the coordinate ρ. A is the only parameter that is a function of coordinate ρ. As we can see above, $\langle |H(k;\rho)|^2\rangle$ for both cases $k \neq m$ and $k = m$ is a function of A^2 which means that $\langle |H(k;\rho)|^2\rangle \propto \mathrm{J}_{|k|}^2(\beta) = \mathrm{J}_{|k|}^2(\mu r_0 \rho)$. Taking advantage of the below integral,

$$\int_0^{\rho_0} \mathrm{J}^2_{|k|}(\mu r_0 \rho)\ \rho\, d\rho = \frac{\rho_0^2}{2}\left\{\mathrm{J}^2_{|k|}(\mu r_0\rho_0) - \mathrm{J}_{|k|-1}(\mu r_0\rho_0)\mathrm{J}_{|k|+1}(\mu r_0\rho_0)\right\}$$

one can find the average of each L^k mode. We have summarized the results in the below table. Note that any conclusion we made here is a function of ratio d/N, so that each of these numbers is not meaningful by itself, and the critical factor is the ratio of these parameters.

Vorticity	$k \neq m$	$V^k = E_0^2(d/N)[1-C_1^2]\ \mathrm{sinc}^2([m-k](d/N)\pi)\ \mathrm{J}^2_{\lvert k\rvert}(\mu r_0\rho_0)$
	$k = m$	$V^m = E_0^2(d/N)^2\frac{e^{-t_1}}{2\pi\sqrt{t}}\ \mathrm{J}^2_{\lvert k\rvert}(\mu r_0\rho_0)\ \{M_1(t_2,t_3) + M_2(t_2,t_3,t_4) + 4h\ [h_1F_1(\epsilon) + h_3F_3(\epsilon) + h_5F_5(\epsilon)]\}$
OAM	$k \neq m$	$L^k = E_0^2(d/N)[1-C_1^2]\ \mathrm{sinc}^2([m-k](d/N)\pi)\ \frac{\rho_0^2}{2}\{\mathrm{J}^2_{\lvert k\rvert}(\mu r_0\rho_0) - \mathrm{J}_{\lvert k\rvert-1}(\mu r_0\rho_0)\mathrm{J}_{\lvert k\rvert+1}(\mu r_0\rho_0)\}$
	$k = m$	$L^m = E_0^2(d/N)^2\frac{e^{-t_1}}{2\pi\sqrt{t}}\ \frac{\rho_0^2}{2}\{\mathrm{J}^2_{\lvert m\rvert}(\mu r_0\rho_0) - \mathrm{J}_{\lvert m\rvert-1}(\mu r_0\rho_0)\mathrm{J}_{\lvert m\rvert+1}(\mu r_0\rho_0)\} \times \{M_1(t_2,t_3) + M_2(t_2,t_3,t_4) + 4h\ [h_1F_1(\epsilon) + h_3F_3(\epsilon) + h_5F_5(\epsilon)]\}$

One important conclusion is the relationship between each vorticity and OAM modes,

$$L^k/V^k = \frac{\rho_0^2}{2}\left\{1 - \mathrm{J}_{|k|-1}(\mu r_0\rho_0)\mathrm{J}_{|k|+1}(\mu r_0\rho_0)/\mathrm{J}^2_{|k|}(\mu r_0\rho_0)\right\}.$$

The significance of this result is that, irrespective of random medium, by measuring one of these properties for a determined circular area, one can find the value of the other one. More precisely, if one performs a phase measurement along a circular closed contour and finds the average of a vorticity mode, the above relation provides the information about the OAM mode, which, in turn, relates to both *phase* and *amplitude* of the field *inside* the contour.

1.3. Special cases

In this appendix we investigate some particular cases. First, we want to find the probability distribution of $|H(k;\rho)|$ in case of no *randomness*. This case is defined by either of these conditions: (i) $d/N \to 1$ or (ii) $\alpha \to 0$. The first case is not applicable because we assumed $d/N \ll 1$ to use the central limit theorem. However, we can consider the case of no variance in phase, which is the case (ii). Under this assumption, for the case of $k \neq m$, both the variance and average go to zero. Below function can describe it

$$\alpha \to 0 \quad \Rightarrow \quad p_{|H(k)|}^{k\neq m} = \lim_{\sigma\to 0} \frac{|H(k;\rho)|}{\sigma^2} \exp\left[-\frac{|H(k;\rho)|^2}{2\sigma^2}\right] = \delta(|H(k;\rho)|) \ ,$$

which is a Dirac delta function centered at zero. Therefore, in the case of $\alpha \to 0$, there is no chance for vorticity to be any value other than m. On the other hand, when $\alpha \to 1$ and $s \to \infty$, then $t_1, t_3, t_4 \to 0$, $t \to (1/4)(N/d)^2$, and $t_2 \to (N/d)^{-1}$. Thus, the probability distribution will be the same as the case of $k \neq m$,

$$p_{|H(k;\rho)|}^{k=m} = \frac{|H(m;\rho)|}{(Nd/2)[\mathrm{J}_{|k|}(\beta)]^2} \exp\left[-\frac{|H(m;\rho)|^2}{(Nd)[\mathrm{J}_{|k|}(\beta)]^2}\right] .$$

In this case, which is the most potent case of randomness, there should not be any difference between the distribution of m and $-m$ vorticities so that the average of total detected vorticity equals zero. Fig. S2 illustrates an example for the case of $m = 5$.

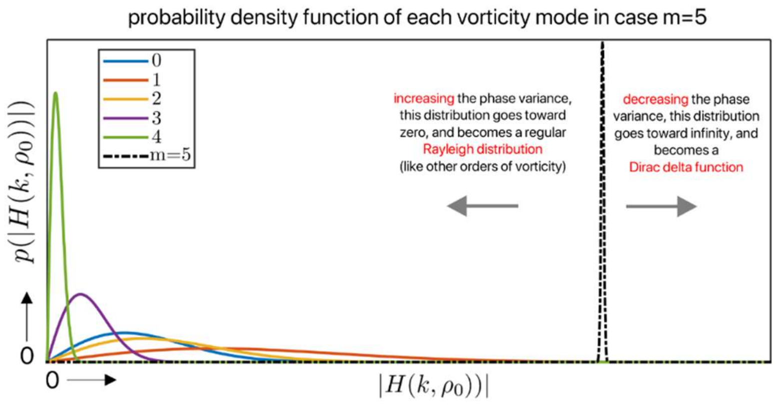

Fig. S2. A general example of probability distributions of different modes of vorticity, for the case of $m = 5$. Here, we just illustrate the modes $0, 1, 2, 3, 4$. Increasing and decreasing the phase variance makes the distribution of initial vorticity (in this example, $m = 5$) to move toward the Rayleigh or Dirac delta distributions, respectively.

2. Numerical simulation

2.1. Details of simulation

The simulation method is based on the Fourier transformation over a perfect optical vortex beam (POV) (see Fig. S3). This beam was generated using a vector (one-dimensional matrix) since POV can be approximated as a thin ring. Under the condition of $N \gg d$, one can assume that the correlation length is invariant along the ring's circumference, just like what we considered in the theoretical model. Therefore, after every d number of discrete units (which are in total N), one new random number was generated (see Fig. S3. (a)). For all the numerical results presented here we used 500 units for variable N while d was varied in the range of 1 to 50 units.

After generating the POV, we apply a Fourier transform to find the scatter field in the far-field regime. Then, one needs to detect the local optical vortices inside the stochastic field. Two conditions were investigated in order to find the optical vortices: (1) being in a local minimum amplitude following by (2) phase wrapping as an integer factor of 2π. Calculation over phase has been done using (see Fig. S3. (c)) [3]

$$M = \frac{1}{2\pi}\sum_{n=1}^{8} \arg[E_n E_{n+1}^*].$$

The parameter M can be any integer. If it is zero, there is no singularity happening in the target pixel, while if it takes any other value, that point will be a phase dislocation. Positive or negative values of M determine the handedness of the local vorticity.

After finding the position of the vortices and their charge (it is usually a charge of ∓ 1 because high order local vortices *are not stable* in a random wavefield), one can find the total charge inside any arbitrary closed contour. Note that to make sure whether the algorithm works well or not, we calculated the superposition of phase gradient over an arbitrary (circular, rectangular, etc.) closed contour in different locations across the random field and compared it with the total charge inside the contour. In this way we insure that they share the same value.

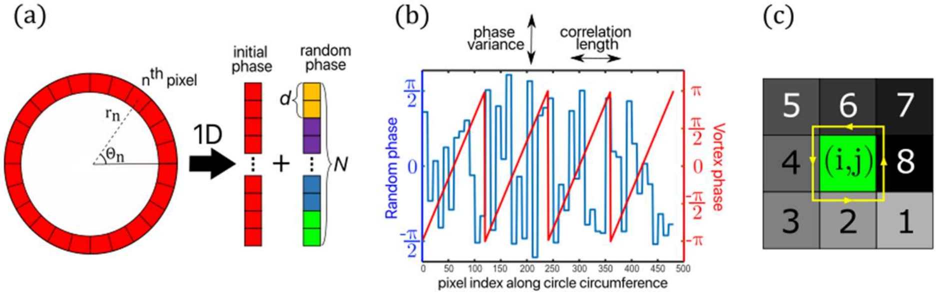

Fig. S3. (a) Schematic of modeling the perfect optical vortex beam (POV) (b) the concept of correlation length and phase variance of randomness in simulation. We have kept the correlation length homogeneous along the ring’s circumference. In this example, the initial vorticity equals 4 meaning that phase is wrapped four times in range $[0,2\pi]$ (c) Detection of the local vortices was done using the superposition of phase gradient over the neighbors of a pixel where local minimum intensity exists.

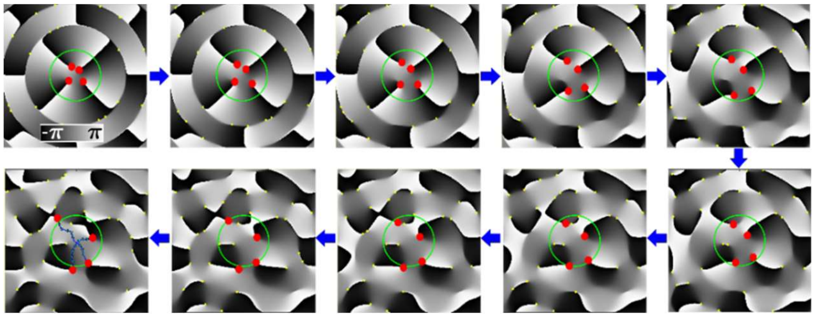

Fig. S4. An example of wandering local vortices which resulted from splitting the initial vorticity (points in red). The movement of these vortices, frame by frame, is continues since the same realization of randomness (with constant correlation length) has been used. The phase variance, however, changes with the same increments at each step. Points in yellow illustrate the local vortices appeared after introducing the perturbation. These vortices can be observed even in the case of Gaussian beam scattering by random media. These figures have been generated by the simulation method described in the previews section.

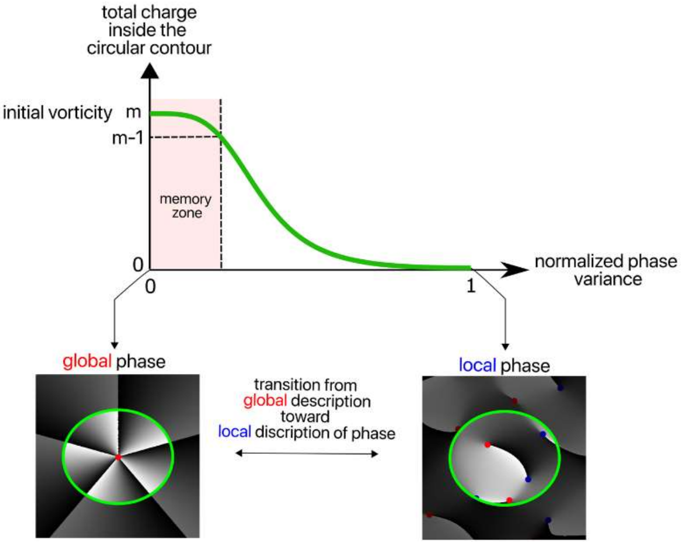

Fig. S5. As shown in the previews figure, local vortices wander from each other until the point where the field becomes homogenous and isotropic stochastic field will be reached. That is the case of fully developed speckle field which is the case of most powerful random distortion. Considering any closed contour, with arbitrary shape and size, which contains the optical axis, the average vorticity detected inside this contour has a general behavior as above plot. The first zone, named "memory area", is the regime of randomness where average vorticity is larger than $m-1$. Operating any interferometric measurement, one can observe global phase pattern based on which some information regarding initial beam can be recovered. In the second area, filed is in transition from an ordered optical field which has experience a weak perturbation toward a strong distortion where field turns into the so-called fully-developed speckle field and global structure for field is meaningless. In fact, field is homogeneous and it can be described just by the distribution of local phase. Note that for above graph, a uniform distribution of randomness was considered such that, in the case of unit normalized phase variance, a fully-speckled pattern would be observed.

2.2. Uniform distribution of randomness

Below is the data calculated for the case of *uniform distribution* ($s \to \infty$) of randomness with phase variation in range $[-\pi, \pi]$. This case corresponding to the strongest randomness, which leads to a fully-developed speckle pattern and to the shortest extent of vortex memory. All axis ranges are the same as the results provides in the main article.

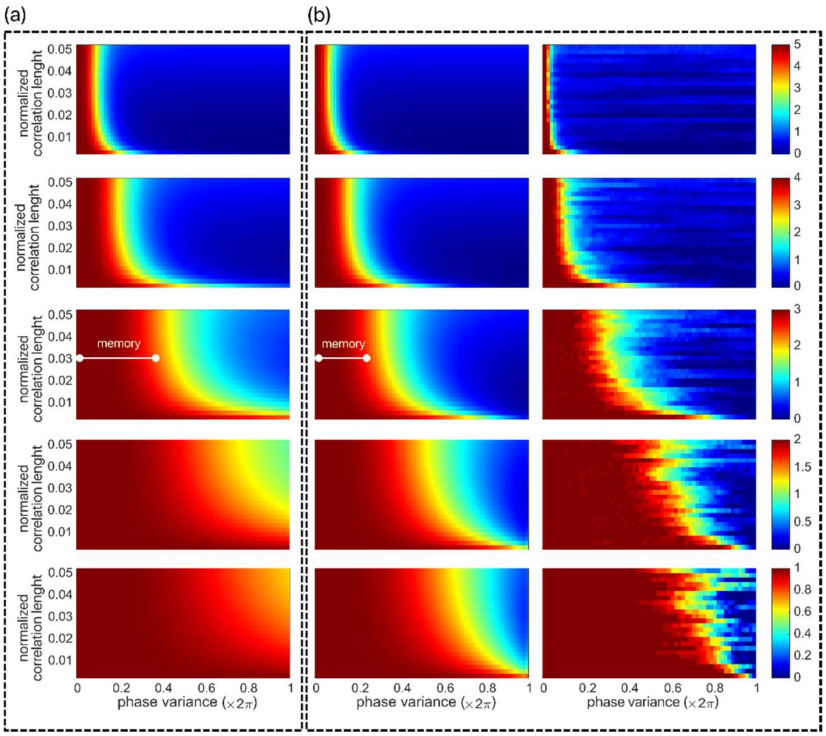

Fig. S6. Effect of randomness on beams with different initial vorticities. The plots illustrate the detected vorticity inside the circular contour with radius $\rho_0 = 2\lambda z/\pi r_0$ as a function of correlation length (d) and phase variance (α) of perturbation.

Panel (a): analytical data for the case of a Gaussian distribution with $s = \alpha \times 0.3\pi$.

Panel (b) analytical data (left column) and numerical data (right column) for the case of uniform distribution of randomness ($s \to \infty$). The simulation results used $N = 500$ and represent the average over 25 different realizations of randomness. Color bars range from zero to the value of initial vorticity.

3. Experiment

3.1. Tunable phase screen (TPS)

The TPS has been designed as the following: two 2-inch Thorlabs 120-ground glass diffusers [4] sandwich a 1.53 series-A refractive index oil [5], where the polished sides of the diffusers face the air. The temperature of the oil was controlled by two HT10KR ceramic ring-shape heaters [6] placed at the two polished sides.

We conducted an independent measurement, prior to the main experiment, to illustrate the TPS capability to impose controllable distortion to an incident field. For this purpose, we used a regular Gaussian beam incident on TPS. We changed the voltage of the heater element and measured the intensity contrast of the emerging speckle field as shown in Fig. S7(b). This test insured that field randomness can be controlled properly to reach a maximum contrast of approximately 0.7. Note that during all experiments, a 10 minutes delay was used to insure that the temperature is homogenous across the glasses.

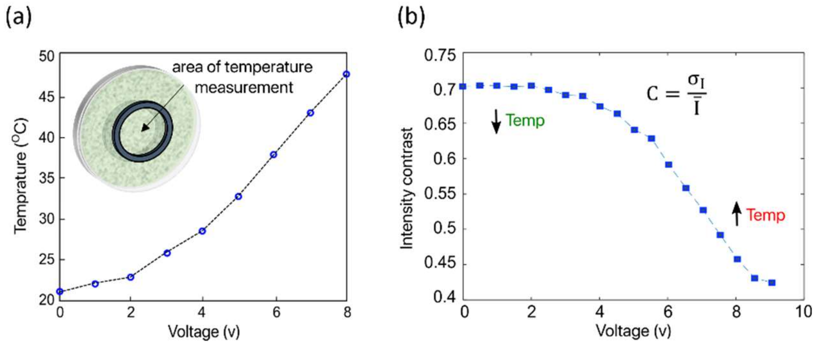

Fig. S7. (a) Measured temperature at the center of phase screen versus the voltage. (b) Intensity contrast for input Gaussian beam. Behavior of the graphs perfectly show the change in the power of randomness of the phase screen.

To find the properties of the ground glass (GG), we measured the roughness along 5 different direction passing through the center and a typical result is illustrated in Fig. S8(a). By evaluating the auto correlation and the roughness distribution, one can assign an effective correlation length and a distribution of phase created by the GG in the presence of oil with a certain refractive index. In the case of our experiment, correlation length was estimated to be $100(\mu m)$ (see Fig. S8(c)).

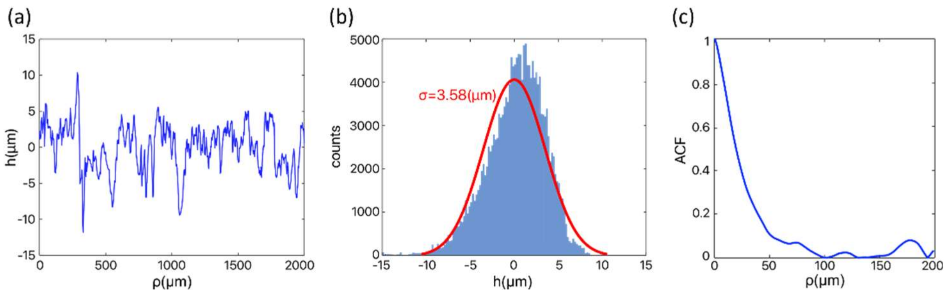

Fig. S8. (a) The surface profile along a line passing through the center of the GG. (b) Distribution of the roughness evaluated from 5 different scans. (c) Auto correlation function of surface fluctuation shown in part (a). based on this plot, we estimated the value of correlation length of GG to be approximately $100(\mu m)$.

Because we use two GGs face-to-face to make this device, we need to characterize the properties of whole system. The probability density function (PDF) of a combination of two GGs is different from each one separately. Indeed, the PDF of the sum of two independent random variables is the convolution of their two PDFs. In the case of our device, PDF regarding GGs are the same; a Gaussian distribution with standard deviation $s_0 \cong 0.36\pi$ (see Fig. S8(b)) (this value is under assumption of $\alpha = 1$). Thus, the convolution of these two same Gaussian distributions results in a Gaussian distribution with standard

deviation $s' = \sqrt{2}s_0^2 \cong 0.6\pi$. The maximum phase range can also be found using the range of Gaussian distribution in Fig. S8(b).

Based on this information and variation of refractive index of the oil versus voltage, the phase variance for the TPS is found to be around 0.7. Note thatThe correlation length was measured before as approximately $100(\mu m)$. Based on this fact that the diameter of POV in the experiment was $\cong 2(mm)$, thus the ratio (which was discussed in first section) is $d/N \cong 0.0159$.

3.2. Details of experiment

This appendix details the phase measurement using two different arrangements. In the first interferometer a spherical wave was used as reference while in the second interferometer the same object wave was used as reference (self-interference). The experimental setup for both measurements is illustrated in Fig. S9.

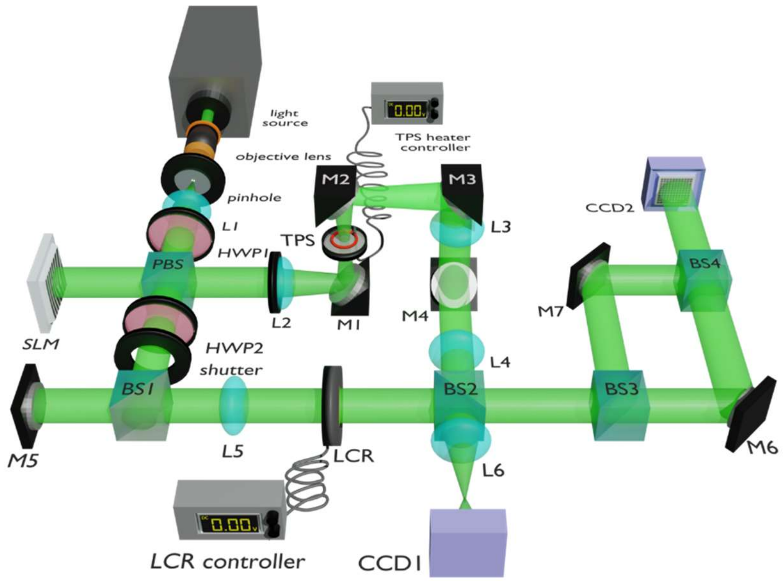

Fig. S9. Experimental setup. All components inside the setup are labeled. In order to label the items, the view has been rotated in comparison to the one in the main text. Note that the aperture at the back of the L2 is an iris diaphragm.

A randomly polarized beam from a Nd:YVO4 laser with wavelength $\lambda = 532(nm)$ is filtered and collimated using an objective, pinhole and lens (L1) with focal distance $f = 10(cm)$. The polarization state of the collimated beam is rotated by a half-wave-plate (HWP1) and split into two orthogonal components by a polarizing beamsplitter (PBS) for the measurement in the first interferometer.

The reflected vertically-polarized component travels toward a spatial light modulator (SLM 512 BNS non-linear system, 512×512 pixels) which works in pure phase mode. By rotating the HWP1, the intensity ratio between the horizontally and vertically polarized components was adjusted to be 1:2, respectively. The reason was to compensate the energy which is lost in reflection by SLM. The SLM is controlled by a computer in real time using MATLAB Software routines to generate the mode-converting holograms. The holograms are blazed phase gratings, designed such that they have the maximum of the energy is in the first diffraction order. Additionally, the holograms are programmed with an extra correction phase factor to ensure the best flatness of the phase in the output Bessel-Gauss beams. The resulting Bessel-Gauss beam with a topological charge value m passes through an iris diaphragm which has been placed to remove the undesired diffraction orders.

Then, we need to generate the perfect optical vortex beam (POV). This beam can be created by axicon [7], width-pulse approximation of the Bessel function [8], liquid crystal spatial light modulators (SLMs) [9], and digital micro-mirror devices (DMDs) [10]. Although the information capacity is inherently reduced, their use in free space and underwater communications has been recently demonstrated [11,12,13,14]. The Bessel-Gauss beam incidents onto a lens (L2) with focal distance $f = 150(mm)$. The perfect optical vortex beam (POV) with approximate diameter of $2(mm)$ is then created on the back focal plane of L2 where the TPS is located.

The emerging speckle pattern is directed towards the Fourier lens (L3) placed at the focal distance $f = 150(mm)$ from the TPS to obtain a non-evolving speckle pattern [15]. The optical field right before the BS2 can be written in Cartesian coordinates as $\boldsymbol{E_2}(x,y) = [E_{2x}, E_{2y}]^{\dagger} = E_{02}(x,y)e^{i\phi_{2(x,y)}}[0,1]^{\dagger}$, where E_{02} and ϕ_2 are the amplitude and phase of the speckle field, respectively.

The transmitted horizontally-polarized component in the PBS passes a half-wave plate (HWP2) orientated at 45 degrees in respect to horizontal, so that it is converted to vertically polarized state of polarization. Then, this vertically-polarized wave-plane is transformed into a spherical wave using a Plano-convex lens L5 with focal distance $150(mm)$. The spherical wave passes through a variable liquid crystal retarder (LCR) to become $\boldsymbol{E_1}(x,y) = [E_{1x}, E_{1y}]^{\dagger} = E_{01}(x,y)\ exp(i\phi_{1(x,y)} + i\delta)[0,1]^{\dagger}$, where E_{01} and ϕ_1 are amplitude and phase, respectively, and parameter δ is an additional phase factor adjusted by LCR. Finally, the transmitted speckle field and the reflected spherical beam are recombined into a single beam by the beamsplitter BS2. The corresponding optical field can be expressed as

$$\boldsymbol{E}_{\boldsymbol{t}(\delta)} = E_{01}e^{i(\phi_1+\delta)}\begin{pmatrix}0\\1\end{pmatrix} + E_{02}e^{i\phi_{2(x,y)}}\begin{pmatrix}0\\1\end{pmatrix}.$$

When the phase factor introduced in retarder is $\delta = \pi/2$, intensity of the interference field is given by

$$I_{t(\pi/2)} = |E_{01}|^2 + |E_{02}|^2 - 2E_{01}E_{02}\ sin(\phi_2 - \phi_1).$$

However, when this parameter is adjusted to $\delta = 0$, the intensity can be written as

$$I_{t(0)} = |E_{01}|^2 + |E_{02}|^2 + 2E_{01}E_{02}\ cos(\phi_2 - \phi_1).$$

Using the two last equations, one can evaluate the phase as

$$tan(\phi_2 - \phi_1) = -\frac{|E_{01}|^2 + |E_{02}|^2 - I_{t(\pi/2)}}{|E_{01}|^2 + |E_{02}|^2 - I_{t(0)}},$$

which is actually the phase difference between the scattered field and the reference field. At this point, we can ensure that the experimental procedure described above, aside from the separate intensities of two arms, $|E_{01}|^2$ and $|E_{02}|^2$, only requires two interferometric measurements of the intensity to obtain the experimental value of the phase $\phi = \phi_2 - \phi_1$, while the four-measurement phase retrieval technique is common. In this way, the phase of optical field is measured fast in a time limited by the modulation frequency of LCR (hundreds of kHz) and the exposure time of camera ($50\ ms$). The lens L4 was used to manipulate the size of speckles, and lens L6 was used for magnifying the interference pattern, because the field around the optical axis (paraxial regime) is desired.

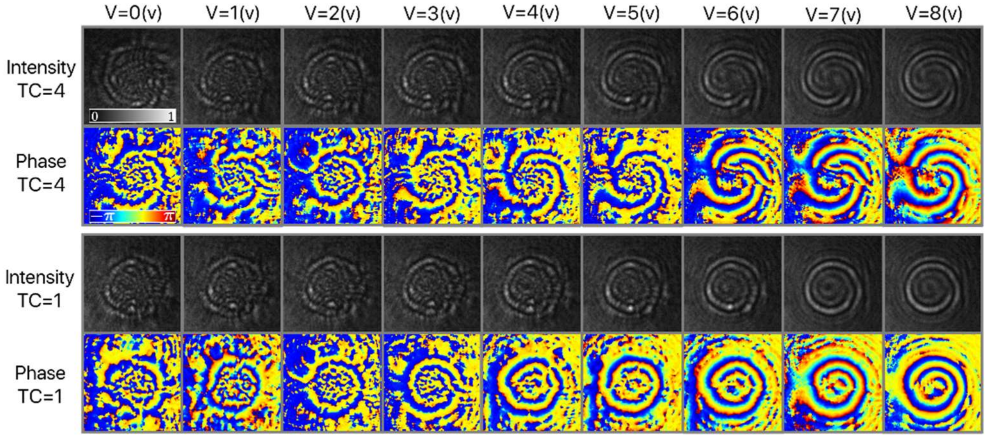

Fig. S10. Interferometric measurements of the intensity pattern with factor $\delta = \pi/2$ and retrieve phase of scattering the perfect optical vortices beams with topological charges $m = 4$ (first and second rows) and $m = 1$ (third and fourth rows).

Experimental results of the interferometric measurements in the first interferometer of the intensity are illustrated in Fig. S10, including the retrieved phase regarding the speckle field of the POV with topological charge $m = 4$ (first and second rows) and $m = 1$ (third and fourth rows) for different voltages applied to the tunable random phase screen. The measurements show that increasing the voltage is equivalent to decreasing the TPS scattering. As a result, the interferometric speckle intensity pattern and the speckle retrieved phase tend to the interferometric results of intensity and phase of the POV with a spherical wave [16].

To quantify the topological charge, we were forced to use the rectangular contours in the Cartesian coordinates. Because of the fact that diffracted POVs have different sizes due to different orders of vorticity, we examined the global topological charge by averaging over a couple of square contours

centered on the optical axis. Furthermore, this procedure alleviates the noise existing in any regular phase measurement.

Next, we measure the gradient of the phase around each closed loop contour to quantitatively determine the number of singularities with topological charge +1 and -1 over it and measure the topological charge as the sum of these singularities. By repeating this process for each rectangular contour from the center to the external position in each retrieved phase pattern, we measure the topological charge using the mean value over the number of contours. As the contour size increases, the evaluated charge tends to saturate. We found that a total of 11 contours over each phase distribution provides an appropriate measurement.

To track the global phase, we used the second interferometer where the object wave interferes with a replica of itself (self-interference). In this configuration the reflected speckle field by BS2 is used as input field for BS3. There, the speckle field is divided into two fields which travel along the upper- and lower-arm of the Mach-Zehnder interferometer to reach the beamsplitter BS4. A relative phase shift α between the two speckle fields is introduced by moving, the mirror M6. Finally, the speckle fields are recombined at the output of the beamsplitter BS4 where the intensity becomes

$$I_{t1} = 2|E_{01}|^2(1 - \cos\alpha).$$

Fig. S11 illustrates and example of measured self-interference intensity of the speckle field for the case of the POVs with topological charge $m = 5$, where voltage varies in range $V = 2(v)$ to $V = 7(v)$. One can observe that the interferometric pattern tends to the typical curved-fork shaped when the TPS randomness decreases. In both arms of setup the camera used is an Andor CMOS.

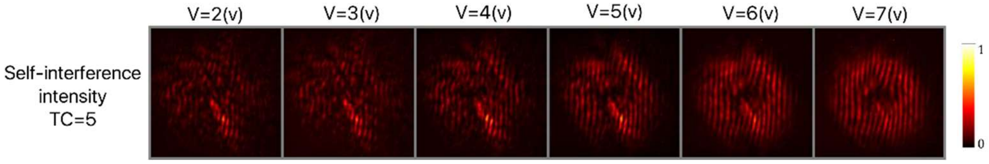

Fig. S11. Self-interference intensity pattern. As it can be observed, by increasing the voltage (decreasing the phase variance), singularity can be observed in the center. Without any phase measurement and with one intensity measurement, one can find the vorticity in the center. That is the reason why this measurement is called "global phase measurement".

References

1. Swartzlander Jr, G.A. and Hernandez-Aranda, R.I., 2007. Optical Rankine vortex and anomalous circulation of light. *Physical review letters*, *99*(16), p.163901.
2. Torner, L., Torres, J.P. and Carrasco, S., 2005. Digital spiral imaging. *Optics express*, *13*(3), pp.873-881.
3. Fried, D.L. and Vaughn, J.L., 1992. Branch cuts in the phase function. *Applied Optics*, *31*(15), pp.2865-2882.
4. Thorlabs, *DG20-120-MD - Ø2", SM2-Mounted N-BK7 Ground Glass Diffuser, 120 Grit.*
5. Liquid, C.L.R.i. *Series A Refractive Index 1.53000*. Available from: https://cargille.com/wp-content/uploads/2018/06/Refractive-Index-Liquid-Series-A-n-1.5300-at-589.3-nm-and-25%C2%B0C.pdf.
6. Thorlabs, R.H. *HT10KR - 10 W Metal Ceramic Ring Heater with 10 kΩ Thermistor, 25 mm OD, 20 mm ID*]. Available from: https://www.thorlabs.com/thorproduct.cfm?partnumber=HT10KR.
7. M. Chen, M. Mazilu, Y. Arita, E. M. Wright and K. Dholakia, *Optics letters,* vol. 38, p. 4919–4922, 2013.
8. J. García-García, C. Rickenstorff-Parrao, R. Ramos-García, V. Arrizón and A. S. Ostrovsky, *Optics letters,* vol. 39, p. 5305–5308, 2014.
9. S. Fu, T. Wang and C. Gao, *Applied optics,* vol. 55, p. 6501–6505, 2016.
10. Y. Liu, Y. Ke, J. Zhou, Y. Liu, H. Luo, S. Wen and D. Fan, *Scientific reports,* vol. 7, p. 1–8, 2017.
11. F. Zhu, S. Huang, W. Shao, J. Zhang, M. Chen, W. Zhang and J. Zeng, *Optics Communications,* vol. 396, p. 50–57, 2017.
12. W. Shao, S. Huang, X. Liu and M. Chen, *Optics Communications,* vol. 427, p. 545–550, 2018.
13. C. Yang, Y. Lan, X. Jiang, H. Long, J. Hou and S. Chen, *Optics Communications,* vol. 472, p. 125879, 2020.
14. Z. Hu, H. Liu, J. Xia, A. He, H. Li, Z. Du, T. Chen, Z. Li and Y. Lü, *Applied Optics,* vol. 59, p. 9956–9962, 2020.
15. Acevedo, C.H., Torres-Moreno, Y. and Dogariu, A., 2019. Spatial intensity correlations of a vortex beam and a perfect optical vortex beam. *JOSA A*, *36*(4), pp.518-525.
16. J. Wang, A. Cao, M. Zhang, H. Pang, S. Hu, Y. Fu, L. Shi, and Q. Deng, 2016. Study of Characteristics of Vortex Beam Produced by Fabricated Spiral Phase Plates," *IEEE Photonics Journal*, *8(2)*, pp. 1-9, Art no. 6500209.
